# Two high-risk susceptibility loci at 6p25.3 and 14q32.13 for Waldenström macroglobulinemia

**DOI:** 10.1038/s41467-018-06541-2

**Published:** 2018-10-10

**Authors:** Mary L. McMaster, Sonja I. Berndt, Jianqing Zhang, Susan L. Slager, Shengchao Alfred Li, Claire M. Vajdic, Karin E. Smedby, Huihuang Yan, Brenda M. Birmann, Elizabeth E. Brown, Alex Smith, Geffen Kleinstern, Mervin M. Fansler, Christine Mayr, Bin Zhu, Charles C. Chung, Ju-Hyun Park, Laurie Burdette, Belynda D. Hicks, Amy Hutchinson, Lauren R. Teras, Hans-Olov Adami, Paige M. Bracci, James McKay, Alain Monnereau, Brian K. Link, Roel C. H. Vermeulen, Stephen M. Ansell, Ann Maria, W. Ryan Diver, Mads Melbye, Akinyemi I. Ojesina, Peter Kraft, Paolo Boffetta, Jacqueline Clavel, Edward Giovannucci, Caroline M. Besson, Federico Canzian, Ruth C. Travis, Paolo Vineis, Elisabete Weiderpass, Rebecca Montalvan, Zhaoming Wang, Meredith Yeager, Nikolaus Becker, Yolanda Benavente, Paul Brennan, Lenka Foretova, Marc Maynadie, Alexandra Nieters, Silvia de Sanjose, Anthony Staines, Lucia Conde, Jacques Riby, Bengt Glimelius, Henrik Hjalgrim, Nisha Pradhan, Andrew L. Feldman, Anne J. Novak, Charles Lawrence, Bryan A. Bassig, Qing Lan, Tongzhang Zheng, Kari E. North, Lesley F. Tinker, Wendy Cozen, Richard K. Severson, Jonathan N. Hofmann, Yawei Zhang, Rebecca D. Jackson, Lindsay M. Morton, Mark P. Purdue, Nilanjan Chatterjee, Kenneth Offit, James R. Cerhan, Stephen J. Chanock, Nathaniel Rothman, Joseph Vijai, Lynn R. Goldin, Christine F. Skibola, Neil E. Caporaso

**Affiliations:** 1 Division of Cancer Epidemiology and Genetics, National Cancer Institute, Bethesda, 20892 MD USA; 2 Department of Epidemiology, School of Public Health and Comprehensive Cancer Center, University of Alabama at Birmingham, Birmingham, 35233 AL USA; 3 Department of Health Sciences Research, Mayo Clinic, Rochester, 55905 MN USA; 4 Cancer Genomics Research Laboratory, Leidos Biomedical Research, Inc., Frederick National Lab for Cancer Research, Frederick, 20877 MD USA; 5 Centre for Big Data Research in Health, University of New South Wales, Sydney, 2052 NSW Australia; 6 Department of Medicine, Solna Karolinska Institutet, Stockholm, 17176 Sweden; 7 Hematology Center, Karolinska University Hospital, Stockholm, 17176 Sweden; 8 Channing Division of Network Medicine, Department of Medicine, Brigham and Women’s Hospital and Harvard Medical School, Boston, 02115 MA USA; 9 Department of Pathology, University of Alabama at Birmingham, Birmingham, 35233 AL USA; 10 Department of Health Sciences, University of York, York, YO10 5DD UK; 11 Tri-Institutional Training Program in Computational Biology and Medicine, Weill Cornell Graduate College, New York, 10021 NY USA; 12 Cancer Biology and Genetics Program, Memorial Sloan Kettering Cancer Center, New York, 10065 NY USA; 13 Department of Statistics, Dongguk University, Seoul, 100-715 Republic of Korea; 14 Epidemiology Research Program, American Cancer Society, Atlanta, 30303 GA USA; 15 Department of Medical Epidemiology and Biostatistics, Karolinska Institutet, Stockholm, 17177 Sweden; 16 Department of Epidemiology, Harvard T.H. Chan School of Public Health, Boston, 02115 MA USA; 17 Institute of Health and Society, Clinical Effectiveness Research Group, University of Oslo, Oslo, NO-0316 Norway; 18 Department of Epidemiology and Biostatistics, University of California, San Francisco, San Francisco, 94118 CA USA; 19 International Agency for Research on Cancer (IARC), Lyon, 69372 France; 20 Epidemiology of Childhood and Adolescent Cancers Group, Inserm, Center of Research in Epidemiology and Statistics Sorbonne Paris Cité (CRESS), Paris, F-94807 France; 21 Université Paris Descartes, Paris, 75006 France; 22 Registry of Hematological Malignancies in Gironde, Institut Bergonié, University of Bordeaux, Inserm, Team EPICENE, UMR 1219, Bordeaux, 33000 France; 23 Department of Internal Medicine, Carver College of Medicine, The University of Iowa, Iowa City, 52242 IA USA; 24 Institute for Risk Assessment Sciences, Utrecht University, Utrecht, 3508 TD The Netherlands; 25 Julius Center for Health Sciences and Primary Care, University Medical Center Utrecht, Utrecht, 3584 CX The Netherlands; 26 Department of Internal Medicine, Mayo Clinic, Rochester, 55905 MN USA; 27 Department of Medicine, Memorial Sloan Kettering Cancer Center, New York, 10065 NY USA; 28 Division of Health Surveillance and Research, Department of Epidemiology Research, Statens Serum Institut, Copenhagen, 2300 Denmark; 29 Department of Medicine, Stanford University School of Medicine, Stanford, 94305 CA USA; 30 Department of Biostatistics, Harvard T.H. Chan School of Public Health, Boston, 02115 MA USA; 31 The Tisch Cancer Institute, Icahn School of Medicine at Mount Sinai, New York, 10029 NY USA; 32 Department of Nutrition, Harvard T.H. Chan School of Public Health, Boston, 02115 MA USA; 33 Service d’hématologie et Oncologie, Centre Hospitalier de Versailles, Le Chesnay, Inserm U1018, Centre pour la Recherche en Epidémiologie et Santé des Populations (CESP), Villejuif, 78157 France; 34 Genomic Epidemiology Group, German Cancer Research Center (DKFZ), Heidelberg, 69120 Germany; 35 Cancer Epidemiology Unit, University of Oxford, Oxford, OX3 7LF UK; 36 MRC-PHE Centre for Environment and Health, School of Public Health, Imperial College London, London, W2 1PG UK; 37 Human Genetics Foundation, Turin, 10126 Italy; 38 Department of Community Medicine, Faculty of Health Sciences, University of Tromsø, The Arctic University of Norway, Tromsø, 9019 Norway; 39 Department of Research, Cancer Registry of Norway, Institute of Population-Based Cancer Research, Oslo, 0379 Norway; 40 Genetic Epidemiology Group, Folkhälsan Research Center and University of Helsinki, Helsinki, 00250 Finland; 41 Westat, Rockville, 20850 MD USA; 42 Department of Computational Biology, St. Jude Children’s Research Hospital, Memphis, 38105 TN USA; 43 Laboratory of Translational Genomics, Division of Cancer Epidemiology and Genetics, National Cancer Institute, Bethesda, 20877 MD USA; 44 Division of Cancer Epidemiology, German Cancer Research Center (DKFZ), Heidelberg, 69120 Baden-Württemberg Germany; 45 Cancer Epidemiology Research Programme, Catalan Institute of Oncology-IDIBELL, L’Hospitalet de Llobregat, Barcelona, 08908 Spain; 46 CIBER Epidemiología y Salud Pública (CIBERESP), Madrid, 28029 Spain; 47 Department of Cancer Epidemiology and Genetics, Masaryk Memorial Cancer Institute and MF MU, Brno, 65653 Czech Republic; 48 EA 4184, Registre des Hémopathies Malignes de Côte d’Or, University of Burgundy and Dijon University Hospital, Dijon, 21070 France; 49 Center for Chronic Immunodeficiency, University Medical Center Freiburg, Freiburg, 79108 Baden-Württemberg Germany; 50 School of Nursing and Human Sciences, Dublin City University, Dublin, 9 Ireland; 51 Bill Lyons Informatics Centre, UCL Cancer Institute, University College London, London, WC1E 6DD UK; 52 Division of Environmental Health Sciences, University of California Berkeley School of Public Health, Berkeley, 94720 CA USA; 53 Department of Immunology, Genetics and Pathology, Uppsala University, Uppsala, 75105 Sweden; 54 Department of Hematology, Rigshospitalet, Copenhagen, 2100 Denmark; 55 Department of Laboratory Medicine and Pathology, Mayo Clinic, Rochester, 55905 MN USA; 56 Department of Epidemiology, Brown University, Providence, 02903 RI USA; 57 Department of Epidemiology, University of North Carolina at Chapel Hill, Chapel Hill, 27599 NC USA; 58 Carolina Center for Genome Sciences, University of North Carolina at Chapel Hill, Chapel Hill, 27599 NC USA; 59 Division of Public Health Sciences, Fred Hutchinson Cancer Research Center, Seattle, 98117 WA USA; 60 Department of Preventive Medicine, USC Keck School of Medicine, University of Southern California, Los Angeles, 90033 CA USA; 61 Norris Comprehensive Cancer Center, USC Keck School of Medicine, University of Southern California, Los Angeles, 90033 CA USA; 62 Department of Family Medicine and Public Health Sciences, Wayne State University, Detroit, 48201 MI USA; 63 Department of Environmental Health Sciences, Yale School of Public Health, New Haven, 06520 CT USA; 64 Division of Endocrinology, Diabetes and Metabolism, The Ohio State University, Columbus, 43210 OH USA; 65 Ontario Health Study, Toronto, M5S 1C6 ON Canada; 66 Department of Biostatistics, Bloomberg School of Public Health, Johns Hopkins University, Baltimore, 21205 MD USA; 67 Department of Oncology, School of Medicine, Johns Hopkins University, Baltimore, 21205 MD USA; 68 Department of Hematology and Medical Oncology, Emory University School of Medicine, Atlanta, 30322 GA USA

## Abstract

Waldenström macroglobulinemia (WM)/lymphoplasmacytic lymphoma (LPL) is a rare, chronic B-cell lymphoma with high heritability. We conduct a two-stage genome-wide association study of WM/LPL in 530 unrelated cases and 4362 controls of European ancestry and identify two high-risk loci associated with WM/LPL at 6p25.3 (rs116446171, near *EXOC2* and *IRF4*; OR = 21.14, 95% CI: 14.40–31.03, *P* = 1.36 × 10^−54^) and 14q32.13 (rs117410836, near *TCL1*; OR = 4.90, 95% CI: 3.45–6.96, *P* = 8.75 × 10^−19^). Both risk alleles are observed at a low frequency among controls (~2–3%) and occur in excess in affected cases within families. In silico data suggest that rs116446171 may have functional importance, and in functional studies, we demonstrate increased reporter transcription and proliferation in cells transduced with the 6p25.3 risk allele. Although further studies are needed to fully elucidate underlying biological mechanisms, together these loci explain 4% of the familial risk and provide insights into genetic susceptibility to this malignancy.

## Abstract (web summary)

Waldenström macroglobulinemia (WM)/lymphoplasmacytic lymphoma (LPL) is a non-Hodgkin-type B cell lymphoma. Here, the authors identify two risk loci for WM/LPL in a two-stage GWAS involving a family-oversampling approach and provide evidence for a functional role of the non-coding SNP rs116446171.

## Introduction

Waldenström macroglobulinemia (WM) is a subset of lymphoplasmacytic lymphoma (LPL) characterized by the presence of an immunoglobulin type M (IgM) monoclonal gammopathy^1^. Together, WM/LPL account for 2% of all non-Hodgkin lymphoma, with an estimated 2330 new cases diagnosed per year in the US^2^. Family history of WM/LPL or related lymphoproliferative disorder is strongly associated with WM/LPL risk^3,4^. Autoimmunity and select infections are also associated with WM/LPL risk^5–8^, and limited data suggest a possible relationship with certain lifestyle and occupational factors^8^, but overall little is known about its etiology. A somatic driver mutation, *MYD88* p.L265P, occurs in most cases of WM^9^. However, germline *MYD88* (myeloid differentiation primary response 88) mutations have not been observed, and despite early promising findings by linkage analysis^10^, no predisposing gene mutations have been conclusively reported to date. Characterizing the genetic factors influencing susceptibility to WM/LPL is an important step toward understanding its etiology. To discover genetic loci for WM/LPL susceptibility, we perform a two-stage genome-wide association study (GWAS) of WM/LPL, leveraging a family-based oversampling approach in the discovery followed by replication in an independent, predominantly non-familial, cohort. Here we report new susceptibility loci at 6p25.3 and 14q32.13 for WM/LPL and provide insights into the genetic etiology of this distinctive B-cell lymphoma.

## Results

### Discovery population, genotyping, and analysis

Oversampling cases with a family history of hematological malignancy, we genotyped 244 WM/LPL cases of European descent (Supplementary Table 1), including 98 unrelated cases (40%) from high-risk families, using the Illumina OmniExpress SNP microarray chip and selected controls previously genotyped on the OmniExpress or Omni2.5^11,12^. Following application of rigorous quality-control metrics, data for 603,492 SNPs with minor allele frequency (MAF) >1% in 217 unrelated cases and 3798 controls of European ancestry remained for analysis (Methods, Supplementary Table 2). A quantile–quantile plot of the association results with genotyped SNPs, adjusted for age, sex, and principal components, revealed enrichment of small *P*-values based on a log-additive genetic model and a small degree of over-dispersion (lambda = 1.05), consistent with other GWAS and due in part to possible polygenic effects^13^ (Supplementary Fig. 1a). Several SNPs at chromosome 6p25.3 reached genome-wide significance with *P*-values between 1.22 × 10^−8^ and 5.64 × 10^−20^ (Supplementary Fig. 2a).

To refine the association signal and identify other regions potentially associated with risk, we imputed common SNPs using the Haplotype Reference Consortium panel^14^. Following imputation and association testing (Supplementary Fig. 1b), the strongest association at 6p25.3 was rs116446171 (*P* = 1.59 × 10^−48^; information score = 0.9985; Supplementary Fig. 2b), a well-imputed SNP between *EXOC2* (Exocyst complex component 2, also known as *Sec5*) and *IRF4* (Interferon regulatory factor 4). A second SNP, rs76106586, was highly significant and in strong linkage disequilibrium (LD; *r*^2^ = 1.0) with rs116446171. Although other SNPs appeared to be strongly associated with risk, conditional association analyses suggest a single signal accounted for the association in this region (Supplementary Table 3). SNPs at chromosome 14q32.13 also reached genome-wide significance, and conditional analyses suggest there may be more than one independent signal at 14q32.13 (Supplementary Fig. 2b; Supplementary Tables 4, 5).

### Independent replication without familial enrichment

**Fig. 1:**
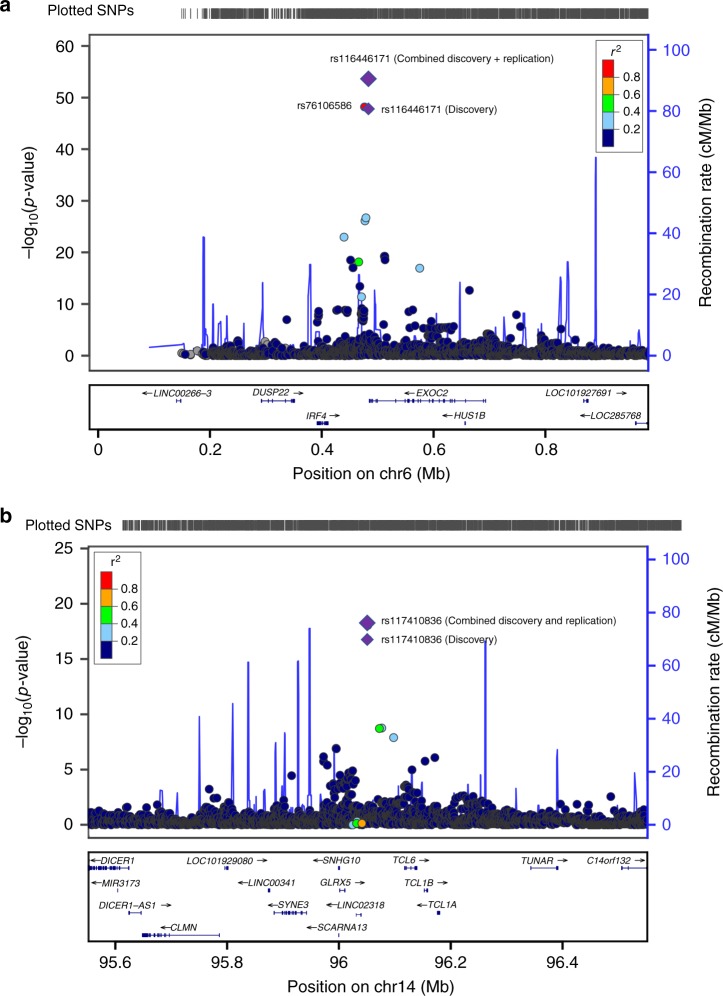
Regional association plots of two SNPs associated with the risk of WM/LPL. **a** Chromosome 6p25.3 (rs116446171) and **b** chromosome 14q32.13 (rs117410836). Shown are the −log_10_ association *P*-values from the discovery log-additive genetic model for all SNPs in the region (dots) and combined discovery and replication fixed-effects analysis (diamonds). The lead SNPs are shown in purple, with results from both the discovery (small diamonds) and combined (large diamonds) analyses. Estimated recombination rates (from 1000 Genomes) are plotted in blue. The SNPs surrounding the most significant SNP are color-coded to reflect their correlation with this SNP. Pairwise *r*^2^ values are from 1000 Genomes European data. Locations of recombination hotspots are depicted by peaks corresponding to the rate of recombination. Genes, position of exons and direction of exons and direction of transcription from UCSC genome browser (http://genome.ucsc.edu) are denoted. Plots were generated using LocusZoom (http://csg.sph.umich.edu/locuszoom)

Eleven SNPs (Supplementary Table 6) with *P* ≤ 5 × 10^−6^ in the discovery analyses were selected for de novo replication in an additional 313 WM/LPL cases, with 24 (8%) reporting a positive family history, and 564 controls of European ancestry (Supplementary Tables 1, 2). The combined analysis of 530 WM/LPL cases and 4362 controls confirmed two distinct loci at chromosome 6p25.3 (rs116446171; odds ratio (OR) = 21.14, 95% confidence interval (CI) = 14.40–31.03, *P* = 1.36 × 10^−54^) and 14q32.13 (rs117410836, OR = 4.90, 95% CI = 3.45–6.96, *P* = 8.75 × 10^−19^), as shown in Table 1 and Fig. 1. Both SNPs were well-imputed in the discovery (information scores >0.96), and technical validation using Taqman or Sanger sequencing showed >99% concordance between imputed and genotyped calls for both SNPs (Supplementary Table 7). The SNPs were genotyped using two different genotyping platforms in the replication, suggesting that our results are not due to platform artifact. Although the effect estimates were attenuated in the replication, suggesting some inflation due to winner’s curse and/or oversampling of familial cases in the discovery, both SNPs replicated with highly statistically significant *P*-values (*P* = 2.54 × 10^−13^ and 1.16 × 10^−5^ for rs116446171 and rs117410836, respectively; Table 1). The effects were similar when the analysis was limited to WM cases (rs116446171: OR = 24.41, 95% CI = 16.46–36.23, *P* = 7.43 × 10^−57^; rs117410836: OR = 5.14, 95% CI = 3.56–7.43, *P* = 2.78 × 10^−18^; Supplementary Table 6). A suggestive second independent signal was observed at 14q32.13 with a directly genotyped SNP (rs179159, *r*^2^ = 0.002, *P* = 4.66 × 10^−7^; Supplementary Table 6), which was slightly attenuated after conditioning on rs117410836 (*P* = 3.16 × 10^−6^). No other locus replicated.

**Table 1:** Association statistics for two independent SNP genotypes and WM/LPL risk

Nearest gene	SNP	Position^a^	Variant Eff^b^/Oth	Stage	EAF controls	Cases/controls *n*/*n*	OR	(95% CI)	*P*-value
6p25.3									
* EXOC2*	rs116446171	484453	G/C	Stage 1	0.0191	217/3798	56.44	(32.89, 96.85)	1.59E−48
				Stage 2	0.0195	312/564	7.71	(4.46, 13.33)	2.54E−13
				Combined		529/4362	21.14	(14.40, 31.03)	1.36E−54
14q32.13									
Intergenic	rs117410836	96051974	C/T	Stage 1	0.0266	217/3798	10.62	(6.17, 18.29)	1.63E−17
				Stage 2	0.0355	306/563	2.81	(1.77, 4.45)	1.16E−05
				Combined		523/4361	4.90	(3.45, 6.96)	8.75E−19

For stages 1 and 2, *P*-values were generated using logistic regression. For the combined stage, the odds ratio and *P-*values were generated using a fixed-effects model controlling for age, gender and genotyping center

*WM* Waldenström macroglobulinemia, *LPL* lymphoplasmacytic lymphoma, *SNP* single-nucleotide polymorphism, *Eff* effect, *Oth* other, *EAF* effect allele frequency, *n* number, *OR* odds ratio, *CI* confidence interval

^a^Genome coordinates are from NCBI human genome GRCh37/human genome (hg) build 19

^b^Variant associated with an effect on risk of WM/LPL

### Risk-variant enrichment in WM/LPL families and heritability

To assess whether the risk variants occurred at a higher than expected frequency within high-risk families, we genotyped the two loci in available affected relatives of familial cases. In families in which the index case had the rs116446171 risk variant, 76% of first-degree relatives with WM or its precursor, IgM monoclonal gammopathy of undetermined significance (MGUS), also carried the risk variant (Supplementary Table 8). Similarly, in families in which the index case had the rs117410836 risk variant, 86% of first-degree relatives with WM or IgM MGUS also carried the risk variant. In both instances, the risk variant frequency in affected relatives exceeded the expected 50% distribution (*P*_binomial_ = 0.01 for rs116446171 and *P*_binomial_ = 0.03 for rs117410836), which is consistent with theoretical models of familial co-segregation^15^. Exploration of heritability in a broader sense, using effect estimated from the replication, indicated that these two loci explain 4% of the familial risk for WM/LPL. When we explored the potential contribution of all common variants to the heritability, we estimated that common SNPs could explain ~25% (95% CI = 15.4–34.5%) of the heritability as a whole, suggesting more common loci are likely to be discovered with larger sample sizes.

### Functional annotation of rs116446171

**Fig. 2:**
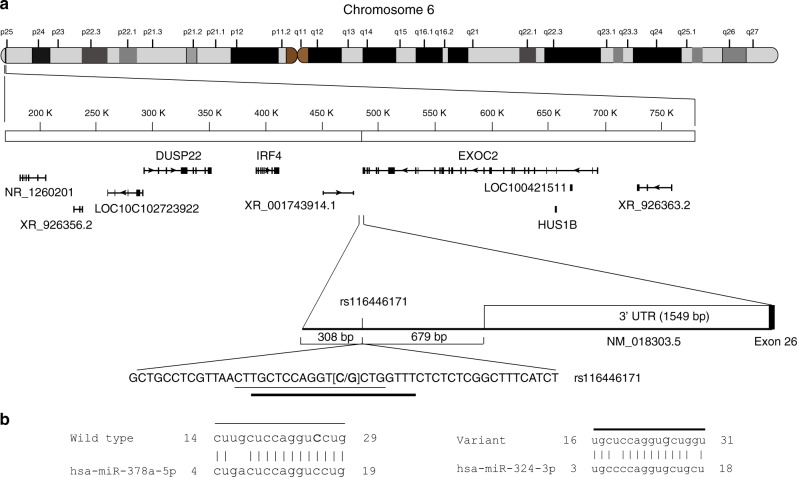
Genomic position and alignments of rs116446171 to miRs. **a** Schematic representation of the position of rs116446171 relative to the 3′UTR of *EXOC2* on chromosome 6 and **b** alignments of rs116446171 wild type and risk variants with the binding sites of microRNAs, miR-378a-5p and miR-324-3p

rs116446171 is located 679 base pairs (bp) downstream of the 3′ untranslated region (UTR) of *EXOC2*, in a region bounded proximally by *IRF4* and *DUSP22* (Dual specificity phosphatase 22) and distally by *EXOC2* (Fig. 2a). To determine whether rs116446171 might be a functional susceptibility variant, we performed in silico analyses that indicated the SNP is located in a region overlapping enhancer histone marks, histone H3 lysine 4 mono-methylation (H3K4me1) and lysine 27 acetylation (H3K27ac), in B-lymphoblastoid cell lines (Supplementary Fig. 3). Analysis of promoter capture Hi-C data^16^ showed that this region interacts with the *IRF4* and, to a lesser extent, *DUSP22* promoters in naïve and total primary B-cells (Supplementary Fig. 3), consistent with reports of long-range enhancer-promoter interactions^17,18^. In silico analyses indicated that the rs116446171 (C) allele (“wild type”) is a predicted binding site for microRNA (miR) miR-378a-5p, and the single-nucleotide change from wild type (C) to risk (G) variant converts the nucleotide sequence to a binding site for a different miR, miR-324-3p (Fig. 2b).

No evidence for significant *cis-*eQTLs (expression quantitative trait loci) within Epstein-Barr virus (EBV)-transformed lymphocytes or whole blood was observed from analysis of Genotype-Tissue Expression Project (GTEx) data (Supplementary Table 9). rs116446171 is not located within the 3′UTR of the annotated transcript of *EXOC2*; however, alternative cleavage and polyadenylation has known capability to alter the length of 3′UTR regions^19^. Cell-type-specific polyadenylation occurs in the immunoglobulin locus and mature B-cells, resulting in transcript isoforms that lead to changes in protein structure^20^. Therefore, we explored in silico data for evidence for an extended UTR for *EXOC2* (Supplementary Fig. 4). Based on published 3′ end-sequencing data performed by various groups^20–22^, we found evidence that rs116446171 could be an enhancer in normal and malignant B-cells as well as other cell types. We then determined whether the SNP can cause changes to the secondary structure of the RNA of *EXOC2*, using RNAfold Webserver^23^ to predict in silico the centroid model with minimum free energy structures and base pair probabilities in the region surrounding rs116446171. As shown in Supplementary Fig. 5, the risk variant is predicted to induce a bulge in the region compared to the other variant. Data from cBioPortal^24^ and COSMIC^25^ for the closest gene, *EXOC2*, indicated a low frequency of somatic mutations in hematopoietic and lymphoid tissues, suggesting that occurrence of age-dependent clonal somatic mosaicism is unlikely to account for our results.

### In vitro functional evaluation of rs116446171

**Fig. 3:**
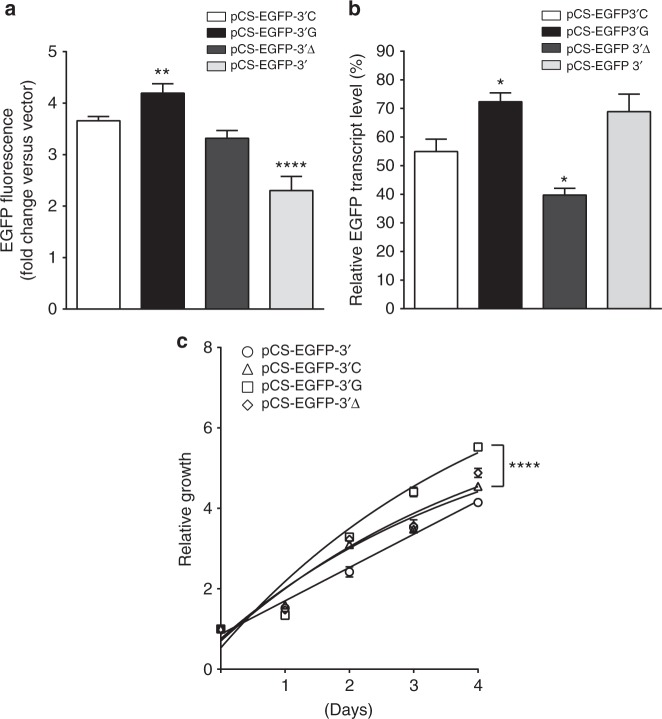
The rs116446171 variants affect reporter activity and cell proliferation. **a** EGFP reporter activity in HEK293T stably transduced cell lines. Cells transduced with the risk variant (G) showed significantly increased fluorescence levels of EGFP compared to the cell lines transduced with the wild type (WT, (C); *P* = 0.012). Cells transduced with the Null (Δ) had decreased EGFP fluorescence (*P* = 0.054, *n* = 14), and cells transduced with the commercial 3′UTR of *EXOC2* showed significantly decreased EGFP fluorescence (*P* < 0.0001, *n* = 14). Data are expressed as mean fold change relative to the cells transduced with the vector, ±standard error of the mean (s.e.m.), *n* = 14 replicates. **P < 0.01, ****P < 0.0001. **b** Quantitative PCR analysis of EGFP transcripts in HEK293T stably transduced cell lines. Significant changes of EGFP mRNA levels were detected in cells harboring the variant allele compared to the cells harboring the wild-type allele (*P* = 0.031). Cells harboring the Null allele had reduced EGFP transcripts levels (*P* = 0.036). Data are expressed as mean % change relative to the endogenous controls, ±s.e.m., *n* = 9 replicates for each experiment. *P < 0.05. **c** Proliferation assay of cells harboring rs116446171, the deletion (Null) of an 18-bp segment centered on rs116446171, and the commercial 3′UTR reporter. The cell line transduced with the variant allele showed significantly increased cell proliferation compared to the cell lines transduced with the *EXOC2* 3′UTR, the WT and the Null. Data are expressed as mean fold change of the cell line in the day seeded, ±s.e.m., *n* = 9 replicates. ****P < 0.0001. All *P*-values were calculated with unpaired *t*-test

To further explore the functional role of the rs116446171 risk variant, we used an enhanced green fluorescent protein (EGFP) reporter assay to estimate transcript levels. In addition to the pCS-EGFP-3′G (risk) construct, we also created pCS-EGFP-3′C (wild type, WT) and pCS-EGFP-3′Δ (null) constructs to use as comparators. Stably transduced HEK293T cell lines were grown for conducting the assays. Cells transduced with the risk allele reporter pCS-EGFP-3′G showed significantly increased EGFP fluorescence compared to the WT, Null, and the commercial EGFP reporters (Fig. 3a). Quantitative PCR analysis showed significantly higher EGFP mRNA levels in cells harboring the risk variant (Fig. 3b), possibly resulting from increased transcription or translational controls such as stability of EGFP mRNA. Furthermore, cells harboring the Null construct had significantly decreased EGFP mRNA levels, suggesting the deleted segment of DNA harboring the SNP might have an important role in the self-maintenance of transcriptional control of EGFP mRNA. We saw no effect on EXOC2 transcript levels (Supplementary Fig. 6). Transduced cells harboring the risk variant showed significantly increased cell proliferation, based on the MTT assay (Methods), compared to cells transduced with the empty, wild type, or null vectors (Fig. 3c).

**Fig. 4:**
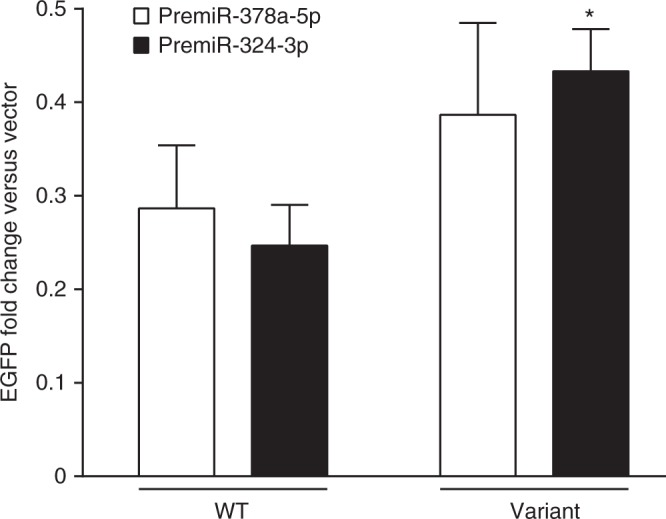
EGFP reporter assay of interactions with microRNAs. Transient transfections with either the PremiR-378a-5p or PremiR-324-3p expression plasmid reduced the EGFP protein expression in cells harboring the wild type or variant allele equally (i.e., resulted in similar fold reductions). Transfection of PremiR-324-3p significantly increased the EGFP fluorescence in the cells harboring the variant allele compared to the wild type (*P* = 0.040). Data are expressed as mean fold change relative to the cells transfected with the pLV-miR vector, ±s.e.m., *n* = 9 replicates. *P*-values were calculated with unpaired *t*-test. *P < 0.05. N.B. the scale of the fold change on the *Y* axis is <1.0

To evaluate the functional effect of the miR-binding site conversion conferred by the risk variant, we created pre-microRNA expression plasmids and transfected cells containing the wild type (C) or risk (G) variant (Methods). Overexpression of either  premiR-378a-5p and premiR-324-3p in cells harboring the wild type or the risk variant decreased EGFP protein fluorescence (Fig. 4). However, no miR-specific effects were observed in the transfected cells overexpressing miR-378a-5p or miR-324-3p, respectively (Supplementary Fig. 7), suggesting that the observed phenotypes in the cells harboring the risk variant might not be due to direct interference of the miRs with translation.

**Fig. 5:**
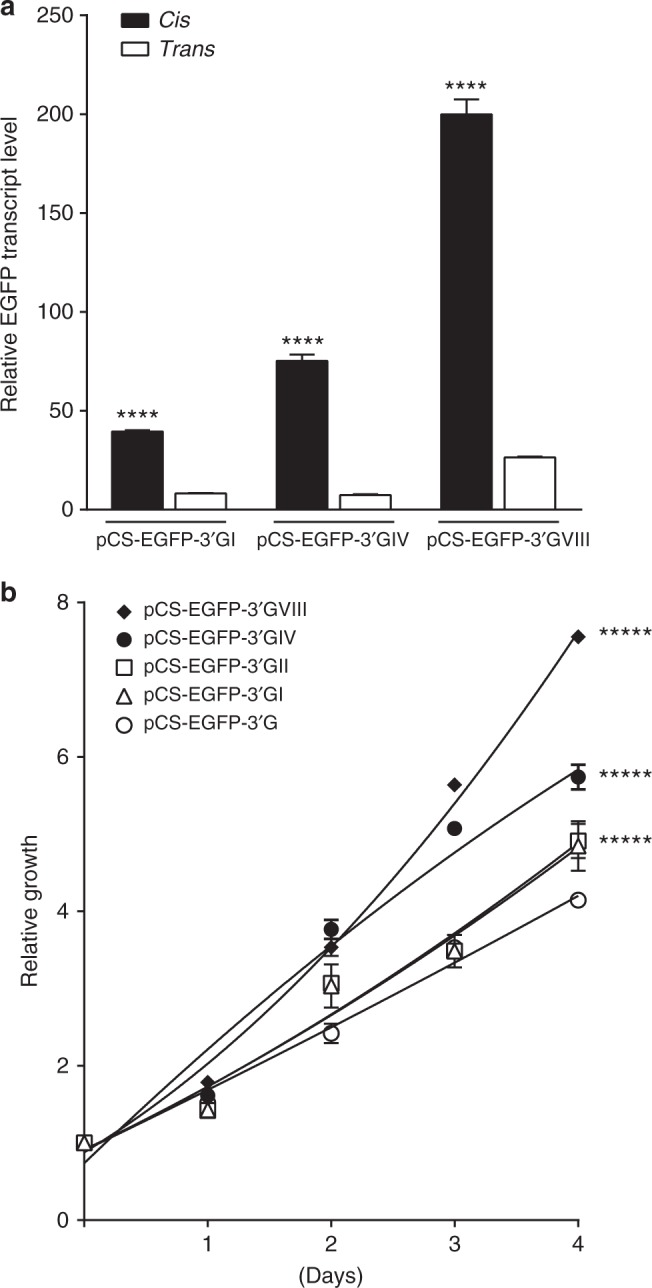
Dose-dependent effect of rs116446171 variants on reporter transcription and cell proliferation. **a** Quantitative PCR analysis of EGFP transcripts in stably transduced cells with tandem repeats of the rs116446171 variant allele. The variant allele was inserted within the *EXOC2* 3′UTR region as a single copy or as two, four and eight repeats in either *cis* or *trans* orientation. Data are expressed as mean fold change of the endogenous controls, ±s.e.m., *n* = 9 replicates. ****P < 0.0001. **b** Proliferation assay of cells transduced with tandem repeats of the variant allele. Cells harboring eight tandem repeats proliferate significantly faster than cells harboring four tandem repeats, and cells harboring four tandem repeats proliferate significantly faster than cells harboring two or one repeat. Data are expressed as mean fold change of the cell line in the day seeded, ±s.e.m., *n* = 9 replicates. ****P < 0.0001. All *P*-values were calculated with unpaired *t*-test

To further investigate the possible role of the miR-324-3p binding site created by the risk variant, we cloned 2, 4, or 8 tandem repeats of the 25-base pair (bp) sequence centered around rs116446171G/C into the 3′UTR of the EGFP reporter, in both *cis* and *trans* orientations (Methods). We observed a proportional increase in EGFP mRNA transcript levels (Fig. 5a) and a similar effect on cellular proliferation (Fig. 5b) from the constructs incorporating increasing numbers of repeats of the rs116446171 risk variant that appeared to be dose-dependent in *cis* but, as expected, not in *trans*. These data suggest rs116446171 alters a binding site for miRNA. The miR-324-3p binding site results in increased gene expression. Previous studies have shown that miRs can influence tumorigenesis through various processes^26,27^. miR-324-3p induces promoter-mediated expression of RelA, a subunit of NF-κB (nuclear factor kappa-light-chain-enhancer of activated B-cells)^28^, and the NF-κB signaling network is important in the pathogenesis of B-cell malignancies, including WM^29,30^. However, the precise mechanism by which non-canonical miR binding might increase WM/LPL risk is unclear.

### Functional annotation of rs117410836

The most significant SNP marker at 14q32.13 (rs117410836) is located near an uncharacterized long non-coding RNA (lncRNA; *LINC02318*), *GLRX5* (glutaredoxin 5), and members of the T-cell leukemia (TCL) gene family (*TCL1A, TCL1B*, and *TCL6*). rs117410836 resides in a repressive chromatin domain with histone H3 lysine 27 trimethylation (H3K27me3) (Supplementary Fig. 3). In silico analyses using Hi-C data suggest that the region containing a linked SNP (rs1150666963; *r*^2^ = 0.75) interacts with the *TCL1A* promoter in total primary B-cells (Supplementary Fig. 3; Methods).

## Discussion

In this study, we used a two-stage genome-wide approach to identify association between genetic variants and the risk of WM/LPL. We oversampled familial WM/LPL cases to enrich for potential susceptibility loci in our discovery stage and used an independent, predominantly non-familial sample to replicate our results. We found two loci at 6p25.3 and 14q32.13 associated with WM/LPL risk in individuals of European ancestry.

Definitive identification of the functional variants and genes remains a major challenge of GWAS. In this study, the most significant SNP, rs116446171 (6p25.3), is identical to that shown to be highly associated with diffuse large B-cell lymphoma in European and East Asian populations^31,32^. While this observation is congruent with data supporting the co-aggregation of B-cell disorders, we cannot yet exclude the possibility that another highly significant linked SNP, such as rs76106586, is responsible for the observed association.

rs116446171 is located in proximity to *EXOC2, IRF4*, and *DUSP22*. These genes and their associated transcriptional programs have been implicated in a variety of lymphoid cancers^33–35^ and are plausible WM/LPL susceptibility genes. IRF4 expression is aberrantly downregulated in WM/LPL^36^, specifically in the plasma cell compartment^37^. IRF4 has a critical role in plasma cell differentiation, class-switch recombination, and germinal center fate decisions^38^, and negatively regulates Toll-like-receptor (TLR) signaling by binding MYD88^39^. DUSP22 modulates immune and inflammatory responses through regulation of MAPK (mitogen-activated protein kinase) signaling^40^ and suppresses IL6/STAT3 (interleukin 6/signal transducer and activator of transcription)-mediated signaling^41^, an important MYD88-independent mechanism for WM cell growth and survival^42^. EXOC2 interacts with Ral (RAS-like proto-oncogene) proteins at the nexus of viral exposure and host immune response^43^ and is critical for cancer cell proliferation, invasion, and metastasis^44,45^; it also interacts with NF-κ pathway constituent, TBK1 (TANK binding kinase 1) to promote tumor cell survival^46^.

The susceptibility locus at 14q32.13, rs117410836, maps most closely to the previously uncharacterized lncRNA, *LINC02318*. LncRNA regulation of transcription, cytokine production and other cellular functions has been implicated in cancer^47^ as well as in the development and function of the innate immune system. Specific lncRNAs influence gene expression programs, including the NF-κB signaling pathway^48^, interact with transcription factors^49^, and have been shown to be induced via the canonical TLR pathway involving MYD88 and NF-κB proteins^50^. Among TCL family members in this region, *TCL1* has been shown to be aberrantly expressed in 73% of WM tumor samples^51^. Dysregulated TCL1 expression in B-cells enhances cell proliferation and survival, leading to cell transformation and mature B-cell tumors through multiple effector mechanisms, including NF-κB activation^52–55^. Limited data are available regarding genetic variation in this region^56^. Thus, additional work is needed to understand the functional implications of variation at this locus and its relationship to WM/LPL.

In conclusion, we performed a GWAS of WM/LPL and identified two independent loci that are associated with the risk of WM/LPL with substantially higher than expected estimated odds ratios for a GWAS of an adult cancer. It is remarkable that the effect size for 6p25.3 is substantively different for WM/LPL compared to other subtypes of NHL. The effect size was especially pronounced in our discovery, where we oversampled familial cases that are more likely to harbor disease variants, but remained highly significant in a predominantly non-familial replication set. The large effect size observed for rs116446171 in 6p25.3 near *IRF4*, *DUSP22*, and *EXOC2* and preliminary in silico and functional evidence suggest we may have discovered an important non-coding variant for WM risk. Familial co-segregation analyses are necessary to understand the implications for familial risk and any warranted clinical application. Additional functional and epidemiological studies are needed to clarify underlying biological mechanisms and to identify additional susceptibility loci that may influence disease risk.

## Methods

### Study approval

Each participating study obtained written informed consent from all participants and approval from its respective human subjects review committee, as follows: CPS-II: Emory University Institutional Review Board; ENGELA: IRB00003888—Comité d’Évaluation Ethique de l’Inserm IRB #1; EPIC: Imperial College London; EpiLymph: International Agency for Research on Cancer; HPFS: Harvard TH Chan School of Public Health Institutional Review Board; Iowa-Mayo SPORE: University of Iowa and Mayo Clinic Institutional Review Board; Mayo CC: Mayo Clinic Institutional Review Board; MSKCC: Memorial Sloan-Kettering Cancer Center Institutional Review Board; NCI Family: NCI Clinical Center Institutional Review Board; NCI-SEER: NCI Special Studies Institutional Review Board; NHS: Partners Institutional Review Board/Brigham and Women’s Hospital; NSW: NSW Cancer Council Ethics Committee; PLCO, NCI Special Studies Institutional Review Board; SCALE: Scientific Ethics Committee for the Capital Region of Denmark and Regional Ethical Review Board in Stockholm (Section 4) IRB#5; UCSF2: University of California San Francisco Committee on Human Research; WHI: Fred Hutchinson Cancer Research Center Institutional Review Board; Yale: Human Investigation Committee, Yale University School of Medicine (See Supplementary Table 1 for study abbreviations).

### Phenotype definition

LPL was defined according to World Health Organization (WHO)^1,57^ criteria. WM was defined according to WHO^1,57^ and required the presence of both an LPL infiltrate in the bone marrow together with a monoclonal immunoglobulin type M (IgM) protein in the serum. In cases where histopathologic criteria for LPL were met but serum protein electrophoresis data were not available, the case was classified as LPL. Diagnoses were validated for all cases by medical and pathology reports. All cases were unrelated. Family history of WM/LPL or other B-cell malignancy (i.e., chronic lymphocytic leukemia (CLL), other non-Hodgkin lymphoma (NHL), Hodgkin lymphoma, or multiple myeloma) was ascertained by self-report, and positive family history was validated in a subset (83%).

### Discovery population

Cases for the stage 1 discovery analysis included 207 participants in a WM family study at the National Cancer Institute (NCI Family)^7^ and 37 cases identified through a case–control study of NHL and CLL at the Mayo Clinic (Mayo CC). The 244 stage 1 cases included 98 reporting a family history of WM/LPL (*n* = 42) or other B-cell malignancy (*n* = 56), 118 cases reporting no family history, and 28 cases with unknown family history. Stage 1 controls (*n* = 3812) were obtained from a previous GWAS of NHL^11^ and included 987 lymphoma-free controls from four US-based studies (National Cancer Institute—Surveillance, Epidemiology, and End Results Interdisciplinary Case-Control Study of Non-Hodgkin lymphoma (NCI-SEER); the Women’s Health Initiative (WHI); a population-based case–control study in Connecticut women (Yale); and Mayo CC) and 2825 cancer-free controls from a study of prostate cancer in the Prostate, Lung, Colon, and Ovarian (PLCO) Cancer Screening Trial^12^ (Supplementary Table 1). Characteristics of the discovery population are shown in Supplementary Table 2.

### Discovery genotyping and quality control

All WM/LPL cases with sufficient DNA (*n* = 244) were genotyped on the Illumina OmniExpress SNP microarray chip at the NCI Cancer Genomics Research Laboratory (CGR). Genotypes were called using Illumina GenomeStudio software, and rigorous quality control metrics were employed to ensure that the resulting data were of high quality (Supplementary Table 10). All data analyses and management were conducted using Genotyping Library and Utilities (GLU), version 1.0 (http://code.google.com/p/glu-genetics/). Specifically, we excluded 15 subjects at this step due to: samples with a call rate of ≤95% (7 cases); insufficient phenotype data (5 cases); and unexpected duplicates (>99.9% concordance; 2 cases, 1 control). Quality control duplicates had >99.9% concordance. Genotype data from the controls were previously generated using the Illumina OmniExpress (Mayo CC, NCI-SEER, WHI, Yale) and the Illumina Omni2.5 (PLCO) chips and underwent the same quality control parameters as cases^11,12^. We assessed ancestry using a set of population informative SNPs^58^ and data from the HapMap CEU, YRI, and ASA populations. We estimated admixture coefficients for each sample using the GLU v1.0 struct.admix module based on the method by Pritchard et al.^59^ and using HapMap^60^ data as the fixed reference populations. Participants with <80% European ancestry were excluded (4 cases, 9 controls; Supplementary Fig. 8). One member of each related pair known to be within three degrees of relatedness by inspection of the pedigree or with estimated pihat >0.4 was excluded (9 cases, 4 controls). After exclusions, all pairwise pihat estimates were <0.08 for cases, and 217 cases and 3798 controls remained for analysis (Supplementary Table 10). For the analysis, SNPs with call rate <95%, Hardy–Weinberg equilibrium *P* < 1 × 10^−6^, minor allele frequency (MAF) < 1%, or previously shown to be analytically uninformative were excluded, yielding 603,492 autosomal SNPs for analysis. To evaluate population substructure, we performed a principal components analysis using the GLU v1.0 struc.pca module, which is comparable to EIGENSTRAT^61^. Plots of the first 5 principal components are shown in Supplementary Fig. 9. We conducted initial association testing assuming a log-additive genetic model, adjusting for age, sex, and two principal components that were found to be significant in the null model.

### Imputation of variants

To allow us to evaluate the genome more comprehensively for SNPs associated with WM, we imputed SNPs in the stage 1 discovery GWAS using the Michigan Imputation Server (https://imputationserver.sph.umich.edu/index.html) and the Haplotype Reference Consortium (HRCr1) panel (https://www.sanger.ac.uk/science/collaboration/haplotype-reference-consortium)^14^ following pre-phasing using SHAPEIT (https://mathgen.stats.ox.ac.uk/genetics_software/shapeit/shapeit.html#home). For imputation, SNPs with MAF < 1% were not excluded. Association testing on the imputed data was conducted using the Frequentist association module in SNPTEST v.2.5.2 (https://mathgen.stats.ox.ac.uk/genetics_software/snptest/snptest.html), assuming dosages for the genotypes, a log-additive model, and adjusting for age, sex and two significant principal components. Tested SNPs with MAF filter <2.5% for cases and <1% for controls or with SNPTEST INFO score filter <0.3 were excluded, resulting in 6,440,053 autosomal SNPs.

### Replication population

An independent stage 2 replication population included 313 WM/LPL cases from the NCI Family study (*n* = 29), Mayo CC/Iowa-Mayo SPORE (*n* = 105), Memorial Sloan Kettering Lymphoproliferative Disorders Study (MSKCC)^62^ (*n* = 64), and ten additional studies^11,12^ (Supplementary Table 1). Among these 313 cases, 24 had a family history of a hematologic malignancy and 105 reported no family history; family history information was reported as unknown or was unavailable for the remainder. The stage 2 controls (*n* = 564) were obtained from Mayo CC/Iowa-Mayo SPORE (*n* = 167), MSKCC (*n* = 302), and eight other studies^11,12^ (Supplementary Table 1). Characteristics of the replication population are shown in Supplementary Table 2.

### SNP selection for replication

After ranking the SNPs by *P-*value and filtering for linkage disequilibrium (LD; *r*^2^ < 0.05), we selected eleven SNPs from the most promising loci identified in stage 1 after imputation with *P* < 5 × 10^−6^ (Supplementary Table 6) for de novo replication in an independent sample of 313 WM/LPL unrelated cases and 564 controls. We chose the most significant imputed and genotyped SNPs at our two top loci (chromosome 6p25.3 and chromosome 14q32.13) and, whenever possible, the most significant SNP (genotyped or imputed) for additional promising loci with *P* < 1 × 10^−6^ in the discovery. Imputed SNPs with an information score ≤0.75 were excluded. Furthermore, only SNPs with MAF > 1% were selected for replication, and no SNPs proceeded to replication if they occurred in regions where they appeared as singletons or obvious artifacts. Of the 11 SNPs selected for replication, five were directly genotyped and the remaining six were imputed in the discovery.

### Replication genotyping and analysis

We conducted genotyping on independent case–control sets in three centers using custom genotyping assays developed for either TaqMan (Applied Biosystems; validated at the NCI CGR) or Sequenom (Sequenom Laboratories; validated at the Mayo Clinic and MSKCC), or Sanger sequencing (for a single SNP, rs117410836, for which a custom TaqMan assay could not be designed). Genotyping was performed by the NCI CGR, the Mayo Clinic and MSKCC and included duplicates for quality control. Following exclusions for genotyping failures and self-reported non-European ancestry, data for 313 cases and 564 controls remained for analysis (Supplementary Table 10). Association testing was conducted assuming a log-additive model, adjusting for age, sex, Ashkenazi ancestry, and genotyping center, which appeared to be an appropriate model for the top SNPs (Supplementary Table 11). The results from the discovery and replication were then combined using a fixed-effects meta-analysis method with inverse variance weighting based on the estimates and standard error from each stage.

### Technical validation

Technical validation was conducted for all SNPs taken forward for replication on a subset of cases (*n* = 213) and controls (*n* = 478) from the discovery. Comparing genotype calls from Taqman assays or Sanger sequencing with genotyped or imputed data from the GWAS showed high concordance (>97%) for all SNPs. Concordance for both genome-wide significant SNPs (rs116446171 and rs117410836) and the secondary signal at chromosome 14 (rs179159) was >99% (Supplementary Table 7).

### Assessment of enrichment among high-risk families

Of the 122 WM/LPL cases in this study reporting a family history, 100 were enrolled in a family study of WM. Among these cases, 21 had the risk variant at rs116446171 or rs117410836 and at least one living relative diagnosed with a relevant B-cell disorder and DNA available for genotyping. To assess whether the risk variants occurred at a higher frequency than expected within high-risk families, we genotyped available affected relatives using the Illumina OmniExpress. After employing rigorous quality control metrics, genotype data were available for 58 relatives, including 32 first-degree relatives and 14 more distantly related relatives with WM, IgM MGUS, or other lymphoproliferative disorders (Supplementary Table 8). For index cases carrying the effect variant at either locus, we computed the frequency with which their affected relatives carried the same effect variant and used the binomial test to determine whether the frequency was greater than expected under the assumption that 50% of first-degree relatives would share the same variant.

### Heritability analysis

To estimate the familial risk explained by these loci for WM/LPL, we estimated the contribution of each independent SNP to the heritability using the equation *h*^2^_SNP_ = *β*^2^2*ƒ*(1 − *ƒ*), where *β* is the log-odds ratio per copy of the risk allele from the replication stage analyses and *ƒ* is the allele frequency, and summed the contributions of all novel SNPs^63^. We then estimated the total heritability based on the estimated relative risks = 24.0 and 20.0 in first-degree relatives for WM and WM/LPL, respectively, from Kristinsson et al.^3^ using the equation derived by Pharoah and colleagues^64^. We calculated the proportion of familial risk explained by dividing the summed contributions of the novel SNPs by the total heritability. To estimate the contribution of all common SNPs to familial risk, we used the method proposed by Yang et al.^65^, which was adapted to dichotomous traits^66^ and implemented in the Genome-wide Complex Trait Analysis (GCTA) software (cnsgenomics.com/software/gcta/). The genetic similarity matrix was estimated from our discovery scan using all genotyped autosomal SNPs with a minor allele frequency >0.01. We used restricted maximum likelihood (REML), the default option for GCTA, to fit the appropriate variance components model, assuming the lifetime risk of WM was 0.00003 to estimate heritability on a liability scale. We then transformed the obtained estimate into a sibling relative risk estimate and estimated the percentage of familial risk explained.

### Functional annotation of rs116446171

To explore whether the rs116446171 SNP was encompassed within *EXOC2* transcripts in any cell type, we analyzed published data from Poly(A)site (http://www.polyasite.unibas.ch/), an annotation tool build using a total of over 400 million reads from 78 3′end sequencing libraries generated with standard 3′ end-sequencing protocols^22^. Poly(A) sites are annotated based on the protein-coding genes and long non-coding RNAs (lncRNAs) contained in the UCSC Basic Table of GENCODE V19. The original data and experimental methods are described by Lianoglou^21^. We analyzed data from the Genotype-Tissue Expression (GTEx) Project (https://www.gtexportal.org/home/) from whole blood, lymphocytes, and EBV-transformed lymphoblastoid cells to determine the presence of *cis* expression quantitative trait loci (eQTLs), adjusted for principal components. We explored possible effects of variation at rs116446171 on secondary RNA structure using RNAfold Server (http://rna.tbi.univie.ac.at/cgi-bin/RNAWebSuite/RNAfold.cgi), a thermodynamic structure prediction tool that predicts secondary minimum free energy structures and base pair probabilities from single RNA or DNA sequences^23^. In parallel, these SNPs were compared to a collection of internally processed epigenetic data sets. Model-based analysis of ChIP-Seq (MACS) package^67^ was used to identify H3K4me1, H3K4me3, and H3K27ac peaks from GSE50893 ChIP-seq data^68^. Reference epigenome data was from the Roadmap Epigenomics Project (http://www.roadmapepigenomics.org); other ChIP-seq and DNase-seq data were from the ENCODE project (https://www.encodeproject.org). Signal tracks (reads per million) were generated using a 200-base pair (bp) sliding window and a step size of 20 bp. To determine potential SNP-gene promoter interactions, we used Capture Hi-C (http://promoter.bx.psu.edu/hi-c/view.php), a method for profiling chromosomal interactions involving targeted regions of interest, such as gene promoters, globally and at high resolution. We used promoter Capture Hi-C data in 17 primary hematopoietic cell types^23^. Regions showing significant chromatin interactions with CHiCAGO (http://regulatorygenomicsgroup.org/chicago) scores ≥5 were downloaded from https://osf.io/u8tzp/.

### Selection of SNP for functional evaluation

Our main finding, rs116446171, is located 679 bp downstream of the 3′UTR of the *EXOC2* gene. Although it is not predicted to be contained in any *EXOC2* transcript, we chose to further evaluate this SNP for possible functional relevance based on (1) prior evidence that this rs116446171 is also the most significant SNP associated with diffuse large B-cell lymphoma^31,32^; (2) the unusually large observed effect size on WM/LPL risk; (3) in silico evidence suggesting an association with regulatory elements in primary B-cells and the lymphoblastoid cell line, GM12878; and (4) the observation that the risk variant might affect a predicted miRNA binding site.

### Cell culture and generation of stable cell lines

HEK293T cells were used because of their reliable growth and transfection profile and suitability for gene expression analysis. HEK293T cells were obtained from American Type Culture Collection (293T ATCC® CRL-3216™; ATCC, Manassas, VA), where they were authenticated by Short Tandem Repeat (STR) profiling. The cells were maintained under standard cell culture conditions at 37 °C in 5% CO_2_ in a humid environment. The culture medium, DMEM, was purchased from Mediatech Inc. (Manassas, VA) and supplemented with 10% FBS from Gemini Bio Products (West Sacramento, CA). Plasmocin Prophylactic (InvivoGen, San Diego, CA) was used in the culture medium to prevent mycoplasma contamination. Lentiviruses were produced by transfection of HEK293T packaging cells with a three plasmid system^69^. To generate stable cell lines, HEK293T cells were seeded into 6-well plates for 24 h before infection with 5 multiplicity of infection (moi) of lentivirus in OptiMEM (Invitrogen; ThermoFisher Scientific, Waltham, MA) in the presence of 8 μg/mL polybrene (Sigma-Aldrich, Inc., St. Louis, MO). After the incubation, medium was replaced with fresh DMEM with 10% FBS for another 24 h before selection in medium containing 1 µg/mL of puromycin, until the control cells were no longer viable. All the stably transduced cell lines were subsequently maintained in medium supplemented with 0.5 µg/mL of puromycin during experimentation.

### Plasmids and site-specific mutagenesis

The *EXOC2* 3′UTR Lenti-reporter-GFP (green fluorescent protein) vector was purchased from Applied Biological Materials Inc. (Richmond, BC, Canada Catalog # MT-h57118). To construct the reporter plasmids with the extended *EXOC2* 3′UTR containing the wild type, variant and deleted sequence (Supplementary Fig. 10a), a wild-type 987 bp fragment PCR-amplified with high Fidelity Platinum PCR SuperMix (Invitrogen) (forward 5′-GGCTTGTCAGGGTTTTCAAG-3′, reverse 5′-CATGCAAAGATGACAAGAGACGG-3′) from human genomic DNA was first cloned into pCRII-TOPO (Invitrogen) and subsequently fused to the *EXOC2* 3′UTR Lenti-reporter-GFP vector with Choo-Choo cloning kit from MCLAB (South San Francisco, CA) to create the construct pCS-EGFP-3′C. For generation of the variant pCS-EGFP-3′G, PCR fragments (forward 5′-GTTGTAATTTACTTGACATTTTTCCCT-3′, reverse 5′-GTTAACTTGCTCCAGCTGCTGGTTT-3′; forward 5′-GTTAACAAACCAGCACCTGGAGCAA-3′, reverse 5′-GCTGGAAATGAAATGCCACT-3′) were amplified with high Fidelity Platinum PCR SuperMix and used to replace the corresponding wild type fragment with Choo-Choo cloning kit. For generation of the deletion construct pCS-EGFP-3′Δ, PCR fragment (forward 5′-CTCGTTAACTTCTCTCTCGGCTTTCATCTAAC-3′, reverse 5′-GCTGGAAATGAAATGCCACT-3′) was amplified and replaced the corresponding wild type fragment with Choo-Choo cloning kit. To construct the empty vector, the commercial *EXOC2* 3′UTR Lenti-reporter-GFP vector was digested with *Eco*RI and *Xho*I and re-ligated with Quick Blunting and Quick Ligation Kits from New England Biolabs (Ipswich, MA). For generation of the tandem repeats of a 25-bp sequence centered around rs116446171 C/G inserted into the *Eco*RI site of the extended *EXOC2* 3′UTR (Supplementary Fig. 10b), PCR primers (forward 5′-GTTAACTTGCTCCAGGTGCTGGTTT-3′, reverse 5′-GTTAACAAACCAGCACCTGGAGCAA-3′) were annealed and ligated into pCRII-TOPO with Quick Blunting and Quick Ligation Kits from New England Biolabs. Following digestion with *Eco*RI, fragments consisting of different tandem repeats were inserted into the EcoRI site of the *EXOC2* 3′UTR. All the clones with *cis* or *trans* orientation and various copy numbers of the tandem repeats were confirmed by sequencing performed by the Heflin Center for Genomic Sciences, University of Alabama at Birmingham.

### Expression and normalization of pre-microRNAs

PremiR-378a-5p (Cat # mir-p227) and PremiR-324-3p (Cat # mir-p193) were expressed in self-inactivated (SIN) lentiviral, pLV-miR vectors purchased from BIOSETTA (San Diego, CA). The control vector (pLV13) was created by insertion of a 118-bp random sequence into the XhoI site of the pLV-miR vector (re-ligation of mir-p227 after deletion of the *Xho*I fragment containing the insert of miR-378a); 4 copies of the tandem repeats in *cis*-orientation were inserted into the *Xho*I site of the pLV-miRNA vector, which served as a locker plasmid (pLV18). All vectors express a rPuro (red fluorescent puromycin-*N*-acetyl-transferase) gene and the measurement of the red fluorescence in cell lines after transfection can be used for normalization. Cells were transfected with the above constructs using lipofectamine to determine the effects of the microRNAs on the EGF reporter. The EGFP fluorescence was measured and normalized against transfection efficiencies in the same cell lines with different microRNA plasmids and normalized against the genomic copy number of EGFP among cell lines transduced with WT, Variant, Null, 3′UTR of *EXOC2* and vectors.

### Cell proliferation assay

Live cells were enumerated at wavelength 570 nm using the MTT assay of mitochondrial enzymatic activity (CellTiter 96 Non-Radioactive Cell Proliferation Assay, Promega Corp., Madison, WI).

### Real-time quantitative RT-PCR

Total RNA was extracted using the RNeasy Mini Kit (Qiagen, Louisville, KY) with on-column DNase digestion. Reverse transcription was performed with Accupower CycleScript RT Premix (Bioneer, Alameda, CA). Real-time quantitative PCR reactions were performed using SYBR green PCR Master Mix from Bio-Rad (Hercules, CA) with EXOC2 primer (forward 5′-GATCCTTCAGCTCATGCACA-3′, reverse 5′-GACTGAGATGGCCCAACACT-3′) and EGFP primer (forward 5′-CAAGATCCGCCACAACATCG-3′, reverse 5′-GACTGGGTGCTCAGGTAGTG-3′). Triplicate reactions for the genes of interest and 4 endogenous controls (ACTB, B2M, GAPDH, and GUSB) were performed separately on the same cDNA samples by using CFX Connect Real-Time System (Bio-Rad). Proprietary primer sets for ACTB, B2M, GAPDH, and GUSB were purchased from RealTimePrimers, LLC (https://www.realtimeprimers.com/). The mean cycle threshold (*C*_t_) was used for the ΔΔ*C*_t_ computations of the relative transcript abundance.

### Normalization for transduction efficiencies of cell lines

Genomic DNAs were purified with Quick-DNA Universal Kit (Zymo Research). Real-time quantitative PCR was performed using SYBR green PCR Master Mix with 3 sets of EGFP primer (Set I: forward 5′-CTTCTTCAAGTCCGCCATG-3′, reverse 5′-ATGTGATCGCGCTTCTCGTT-3′; Set II: forward 5′-CGTAAACGGCCACAAGTTCA-3′, reverse 5′-CTTCATGTGGTCGGGGTAGC-3′; Set III forward 5′-CAAGATCCGCCACAACATCG-3′, reverse 5′-GACTGGGTGCTCAGGTAGTG-3′), while GAPDH was used for the reference gene (Set I: forward 5′-CCCTTCATTGACCTCAACTACATGGT-3′, reverse 5′-GAGGGGCCATCCACAGTCTTCTG-3′; Set II: forward 5′-GTGAAGGTCGGAGTCAACG-3′, reverse 5′-TGAGGTCAATGAAGGGGTC-3′). The ratio of copy number of the integrated reporter gene in cell lines was calculated against the reference gene GAPDH.

### Direct measurement of EGFP and rPuro fluorescence

EGFP and rPuro fluorescence were measured directly with 6-well plates with or without Triton X-100 (1%) treatment using Synergy H1 Hybrid Reader (BioTek, Winooski, VT) Gen5 Microplate Reader and Imager Software (ex/em for GFP: 485/519 nm; for rPuro: 553/593 nm). Fluorescence measurements were performed using the bottom optic, orbital averaging with 2 mm diameter and 20 flashes per well. All the cell lines were cultured in the phenol red free DMEM media (Mediatech, Corning, NY) supplied with 10% FBS to lower the background. All measurements were background subtracted (HEK293T cells with no plasmid transfection) and normalized against the genomic copy number of the EGFP genes.

### In silico miR-binding site prediction

miRBase (http://www.mirbase.org/search.shtml) homologous search with the wild type rs116446171 sequence detected significant homology with the mature microRNA, miR-378a-5p. rs116446171 sequence with a single C to G change eliminates that homology, and instead creates a new homology with the mature microRNA, miR-324-3p.

### Statistical analysis for functional experiments

All the experimental data from cell viability and proliferation assays, EGFP reporter assay, and quantitative PCR analysis were performed by the unpaired *t*-test and fitted with an exponential growth equation of Prism 6 for Windows from GraphPad Software (San Diego CA). Data are shown as means ± the standard error of the mean (s.e.m.) of values obtained for *n* replicates as indicated in the figure legends.

## Disclaimer

The content of this publication does not necessarily reflect the views or policies of the Department of Health and Human Services, nor does mention of trade names, commercial products, or organizations imply endorsement by the U.S. Government.

## Electronic supplementary material

Supplementary Information

## Data Availability

The genotyping dataset generated during this study has been deposited in the dbGaP repository with the accession code phs001284.v1.p1. All other relevant data generated for this study are available upon request from the authors.
