# Supplementary material for: Two high-risk susceptibility loci at 6p25.3 and 14q32.13 for Waldenström macroglobulinemia
Source: Nat Commun. 2018 Oct 10;9:4182. doi: 10.1038/s41467-018-06541-2 (PMC6180091; doi:10.1038/s41467-018-06541-2)
Supplement: Supplementary file 1 — Supplementary Information [file 41467_2018_6541_MOESM1_ESM.pdf]

Two high-risk susceptibility loci at 6p25.3 and 14q32.13 for Waldenström macroglobulinemia

McMaster *et al.*

Supplementary Information

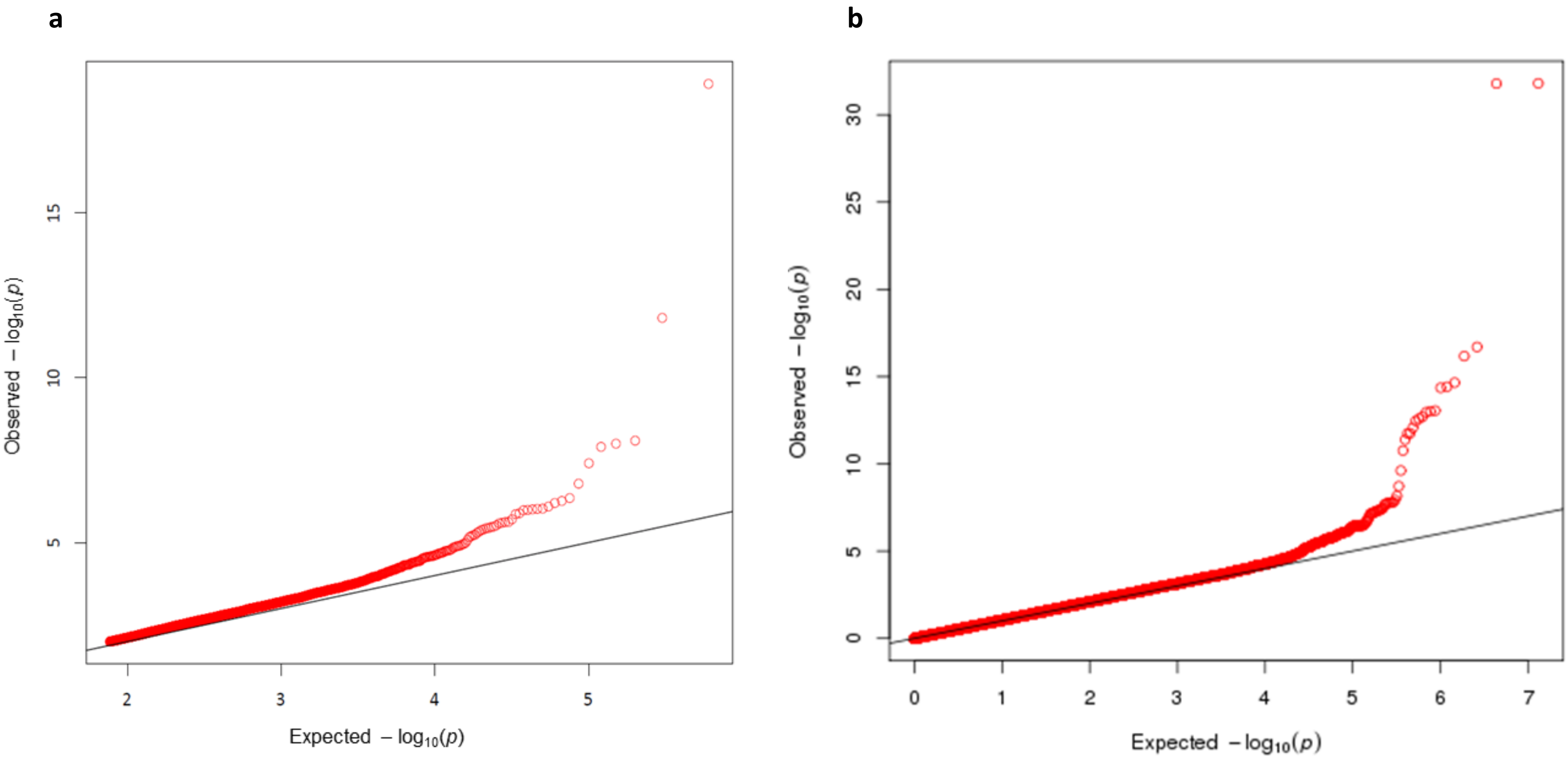

Supplementary Figure 1 | Quantile-quantile (Q-Q) plot of the association results for WM/LPL. Results are shown for Stage 1 discovery genotyping **(a)** and imputation **(b)**. Lambda, λ = 1.05. Note differing scales.

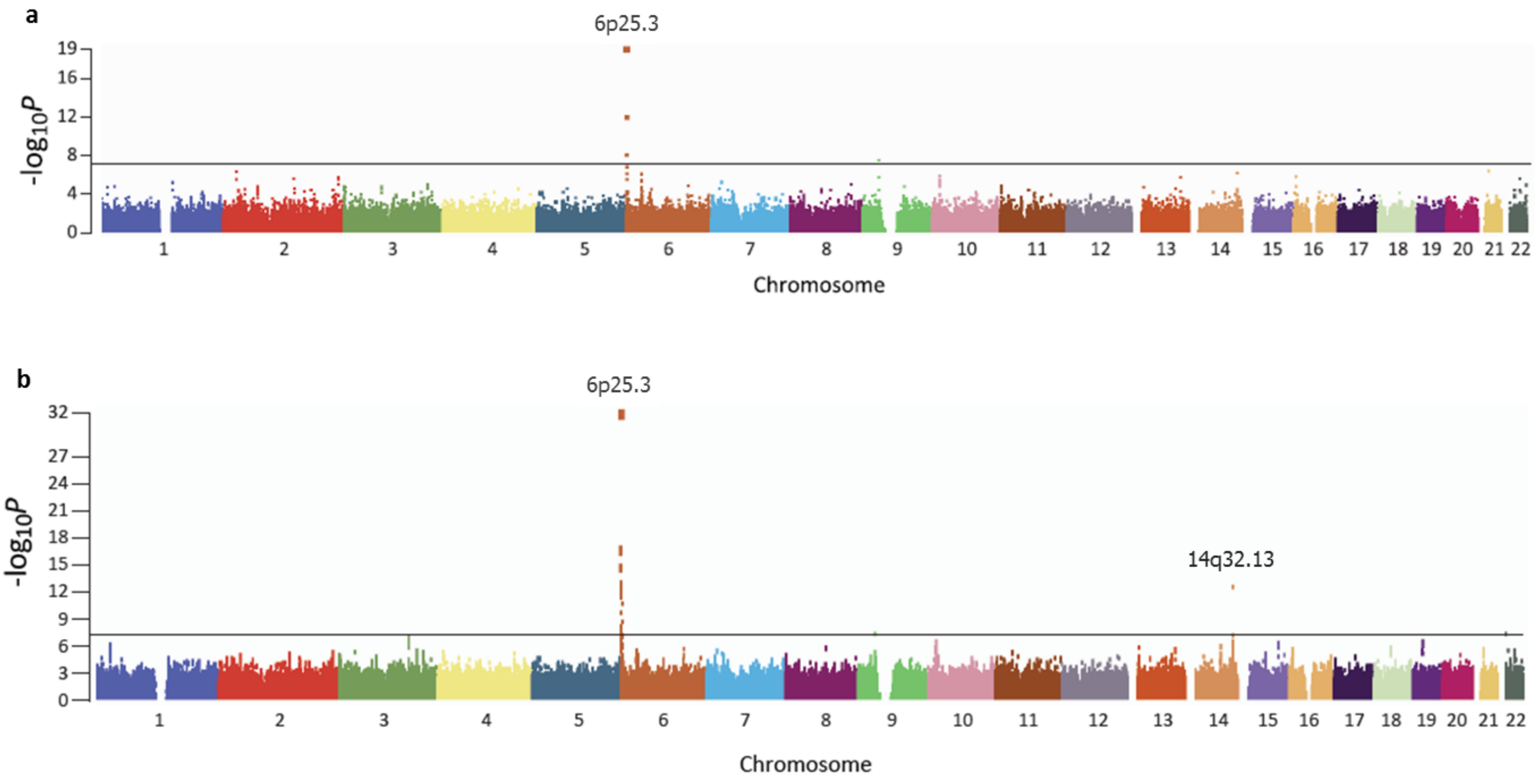

Supplementary Figure 2 | Genome-wide Manhattan plot of the WM/LPL GWAS (Stage 1 discovery). Autosomes are depicted on X axis and the -$\log_{10}$ of *P*-values on Y axis. Results are shown following genotyping (**a**) and imputation (**b**) and association testing using a log-additive model adjusted for age, sex and two principal components found to be significant in the null model.

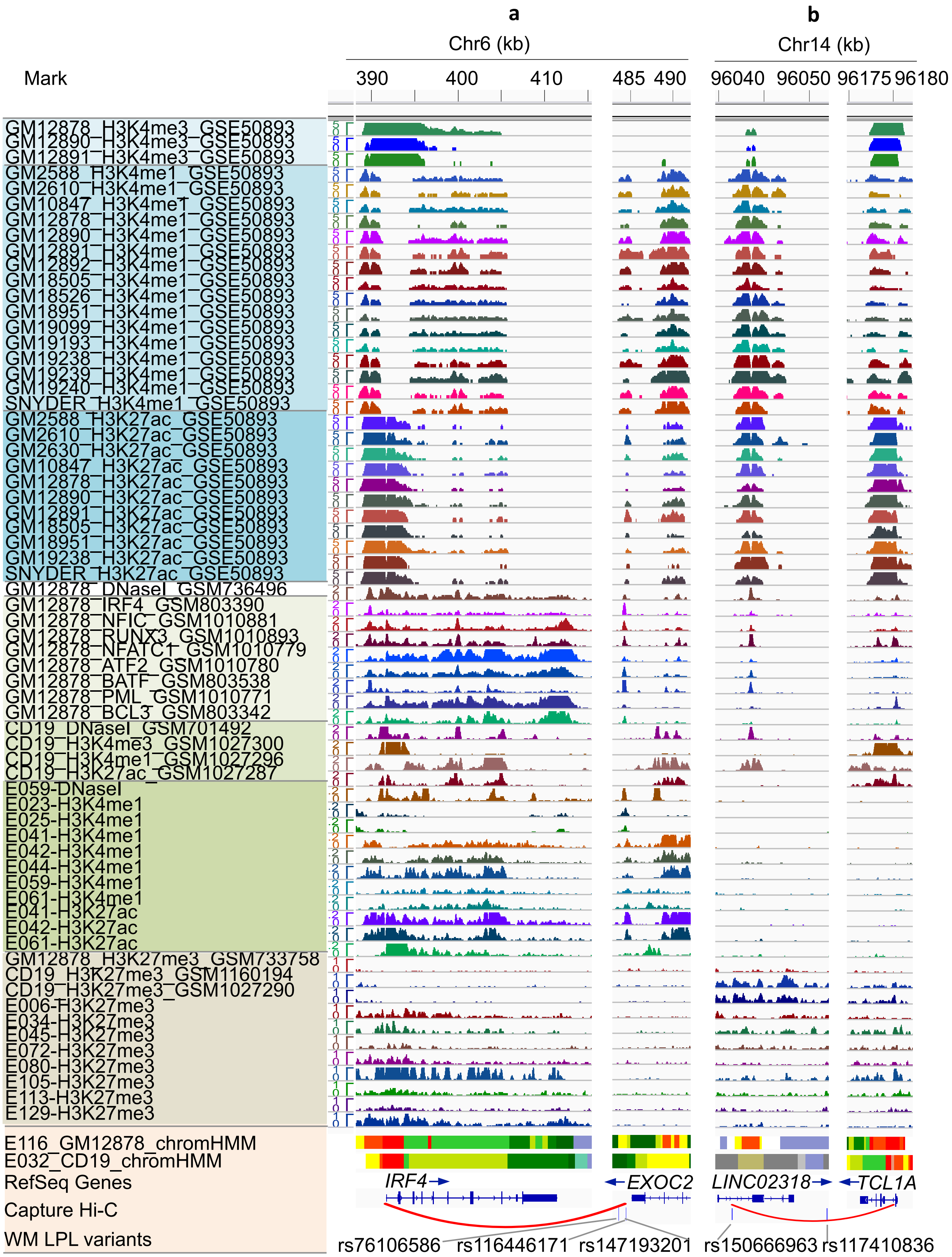
a
b
Chr6 (kb)
Chr14 (kb)
Mark
390
400
410
485
490
96040
96050
96175
96180
GM12878_H3K4me3_GSE50893
GM12890_H3K4me3_GSE50893
GM12891_H3K4me3_GSE50893
GM2588_H3K4me1_GSE50893
GM2610_H3K4me1_GSE50893
GM10847_H3K4me1_GSE50893
GM12878_H3K4me1_GSE50893
GM12890_H3K4me1_GSE50893
GM12891_H3K4me1_GSE50893
GM12892_H3K4me1_GSE50893
GM18505_H3K4me1_GSE50893
GM18526_H3K4me1_GSE50893
GM18951_H3K4me1_GSE50893
GM19099_H3K4me1_GSE50893
GM19193_H3K4me1_GSE50893
GM19238_H3K4me1_GSE50893
GM19239_H3K4me1_GSE50893
GM19240_H3K4me1_GSE50893
SNYDER_H3K4me1_GSE50893
GM2588_H3K27ac_GSE50893
GM2610_H3K27ac_GSE50893
GM2630_H3K27ac_GSE50893
GM10847_H3K27ac_GSE50893
GM12878_H3K27ac_GSE50893
GM12890_H3K27ac_GSE50893
GM12891_H3K27ac_GSE50893
GM18505_H3K27ac_GSE50893
GM18951_H3K27ac_GSE50893
GM19238_H3K27ac_GSE50893
SNYDER_H3K27ac_GSE50893
GM12878_DNaseI_GSM736496
GM12878_IRF4_GSM803390
GM12878_NFIC_GSM1010881
GM12878_RUNX3_GSM1010893
GM12878_NFATC1_GSM1010779
GM12878_ATF2_GSM1010780
GM12878_BATF_GSM803538
GM12878_PML_GSM1010771
GM12878_BCL3_GSM803342
CD19_DNaseI_GSM701492
CD19_H3K4me3_GSM1027300
CD19_H3K4me1_GSM1027296
CD19_H3K27ac_GSM1027287
E059-DNaseI
E023-H3K4me1
E025-H3K4me1
E041-H3K4me1
E042-H3K4me1
E044-H3K4me1
E059-H3K4me1
E061-H3K4me1
E041-H3K27ac
E042-H3K27ac
E061-H3K27ac
GM12878_H3K27me3_GSM733758
CD19_H3K27me3_GSM1160194
CD19_H3K27me3_GSM1027290
E006-H3K27me3
E034-H3K27me3
E045-H3K27me3
E072-H3K27me3
E080-H3K27me3
E105-H3K27me3
E113-H3K27me3
E129-H3K27me3
E116_GM12878_chromHMM
E032_CD19_chromHMM
RefSeq Genes
Capture Hi-C
WM LPL variants
IRF4
EXOC2
LINC02318
TCL1A
rs76106586
rs116446171
rs147193201
rs150666963
rs117410836

Supplementary Figure 3 | Epigenetic features at two WM LPL risk loci.

(a) The tag SNP rs116446171 and its linked INDEL rs147193201 (R^2=0.96) on chromosome 6 were found to reside in an enhancer with histone marks, histone H3 lysine 4 mono-methylation (H3K4me1) and lysine 27 acetylation (H3K27ac) but no lysine 4 trimethylation (H3K4me3) in lymphoblastoid cell lines. This enhancer was missed in B cells but identified in seven other cell types (E023 to E061). The risk locus showed long-range interaction, indicated by red line, with the promoter of *IRF4* that is 90,702 base pairs (bp) away in five of the 17 blood cell types including naive B, total B, activated total CD4+ T, non-activated total CD4+ T, and naive CD4+ T cells based on capture Hi-C data[1]. **(b)** The tag SNP rs117410836 on chromosome 14 resides in a repressed Polycomb or heterochromatin region in E032 (CD19) and E116 (GM12878). It overlapped with lysine 27 trimethylation (H3K27me3) peaks from several cell types but not with any of the three active histone modifications. No target gene promoter was found for this SNP. Nevertheless, the linked SNP rs150666963 (R^2=0.75; 10,455 bp away) was found to interact with the promoter of *TCL1A* (137,477 bp away) in total B cells. Capture Hi-C data in 17 primary hematopoietic cell types were from Jin *et al*[2]. Regions showing significant chromatin interactions with CHiCAGO[3] scores ≥5 were downloaded from https://osf.io/u8tzp/. Model-based analysis of ChIP-Seq (MACS) package[4] was used to identify H3K4me1, H3K4me3, and H3K27ac peaks from GSE50893 ChIP-seq data[5]. Reference epigenome data were from the Roadmap Epigenomics Consortium[6]; other ChIP-seq and DNase-seq data were from the ENCODE project[7].

ChromHMM

1 Active TSS
2 Flanking active TSS
3 Transcription at gene 5′ and 3′
4 Strong transcription
5 Weak transcription
6 Genic enhancers
7 Enhancers
8 ZNF genes + repeats
9 Heterochromatin
10 Bivalent/poised TSS
11 Flanking bivalent TSS/Enh
12 Bivalent enhancer
13 Repressed Polycomb
14 Weak repressed Polycomb
15 Quiescent/low

Standardized Epigenome name

E006, H1 Derived Mesenchymal Stem Cells
E023, Mesenchymal Stem Cell Derived Adipocyte Cultured Cells
E025, Adipose Derived Mesenchymal Stem Cell Cultured Cells
E032, Primary B cells from peripheral blood
E034, Primary T cells from peripheral blood
E041, Primary T helper cells PMA-I stimulated
E042, Primary T helper 17 cells PMA-I stimulated
E044, Primary T regulatory cells from peripheral blood
E045, Primary T cells effector/memory enriched from peripheral blood
E059, Foreskin Melanocyte Primary Cells skin01
E061, Foreskin Melanocyte Primary Cells skin03
E072, Brain Inferior Temporal Lobe
E080, Fetal Adrenal Gland
E105, Right Ventricle
E113, Spleen
E116, GM12878 Lymphoblastoid Cells
E129, Osteoblast Primary Cells

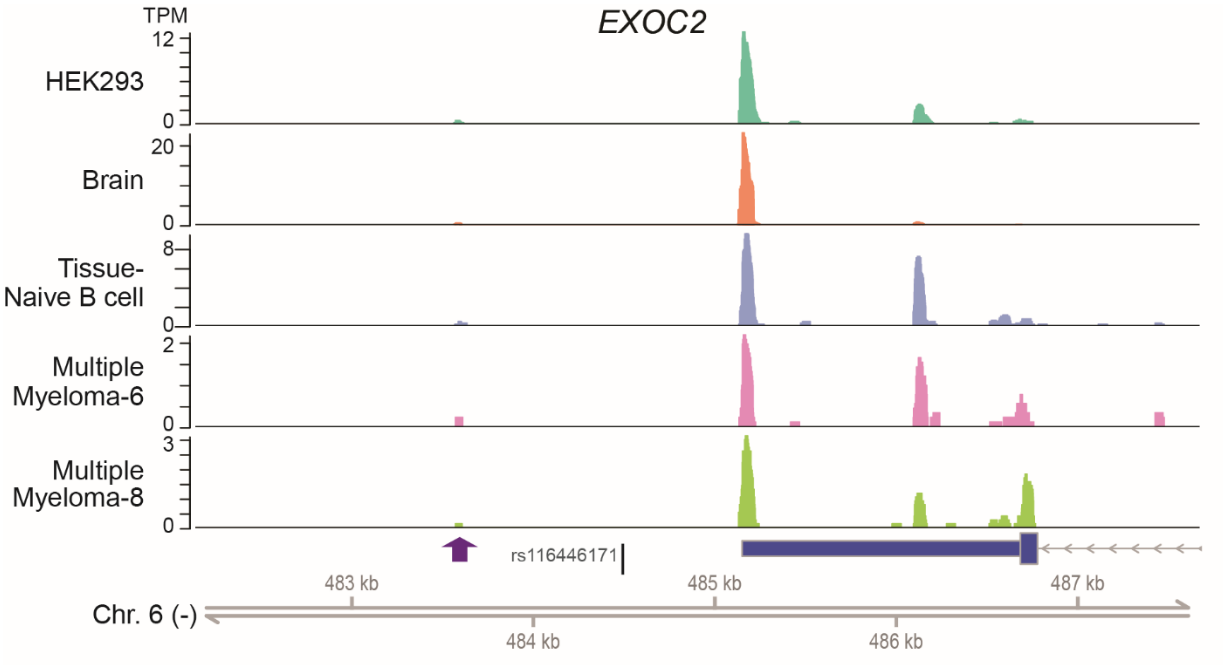

Supplementary Figure 4 | Polyadenylation analysis suggests a potential extended 3'-UTR for *EXOC2*. The 3'-seq (tags per million (TPM)) and RNA-seq (read coverage) tracks showing expression of the intronic polyadenylation (upward pointing arrow) and full length mRNA isoforms of EXOC2. The position of the SNP rs116446171 between the extended UTR and the annotated UTR (heavy bar) is also shown. See Methods for details.

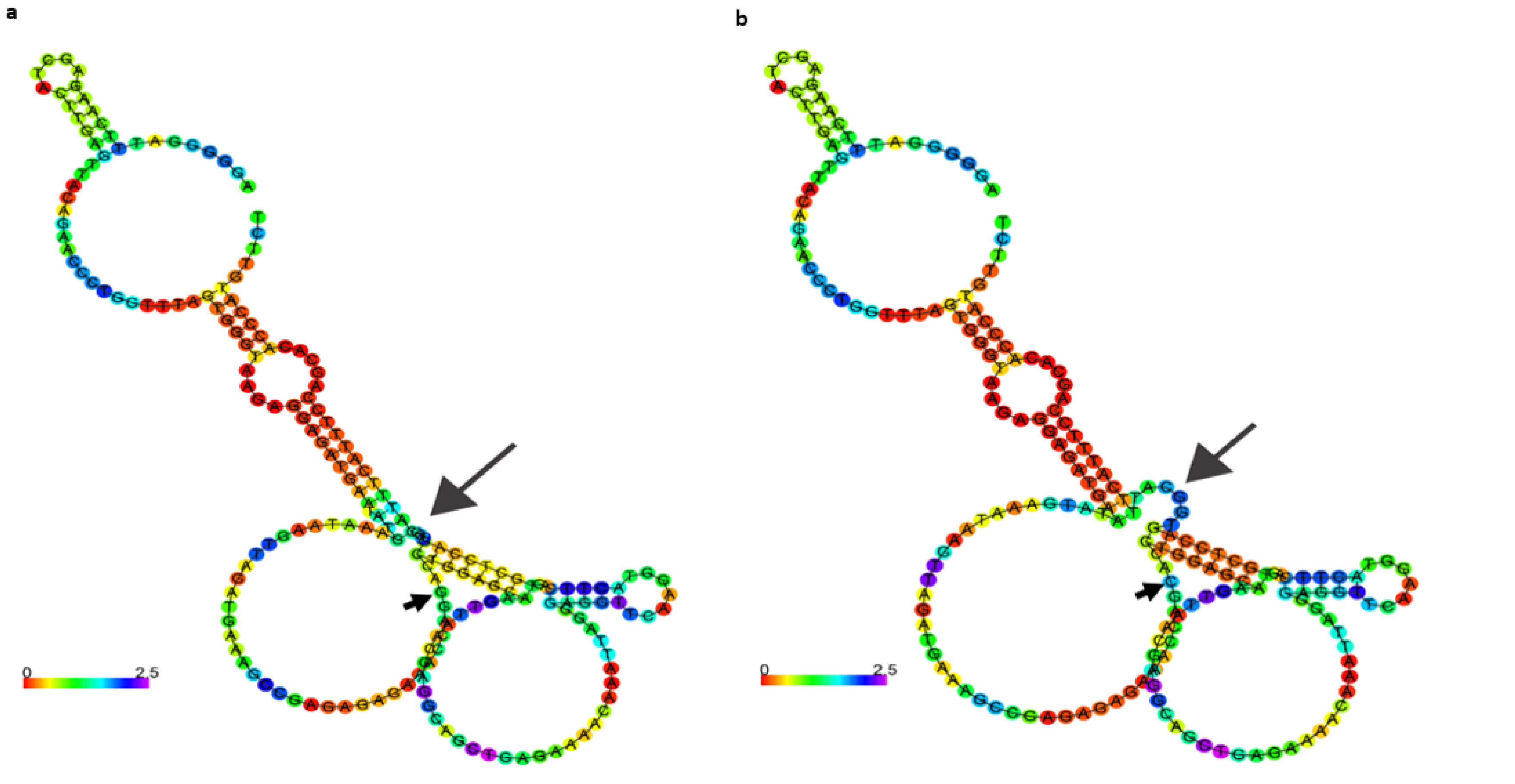

Supplementary Figure 5 │ Predicted effect of the rs116446171 risk allele on secondary RNA structure in the vicinity of the *EXOC2* 3'-UTR by RNAfold Server analysis. Reverse complement sequence is shown. The large arrows indicate the predicted RNA structural change. The small arrows indicate the location of the rs116446171 SNP 'wild-type' (**a**) and risk (**b**) alleles. The color scale is reliability annotation from positional entropy, where red indicates low entropy and high reliability and violet, high entropy and low reliability. See Methods for details.

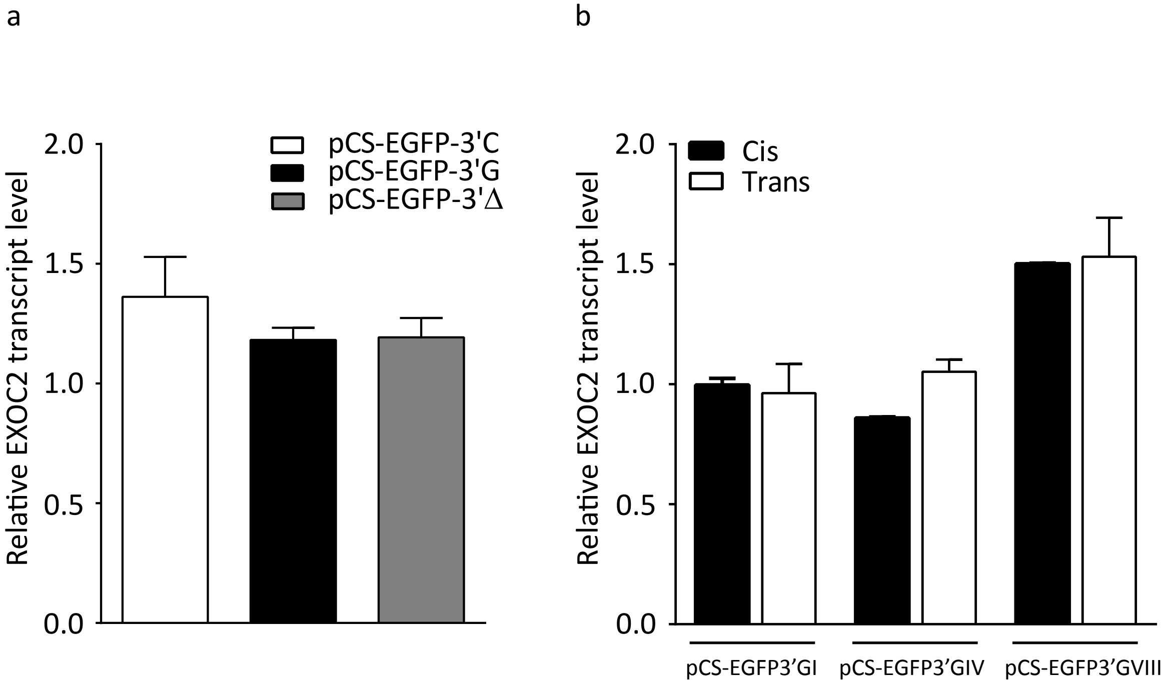

Supplementary Figure 6 | The rs116446171 risk allele does not affect canonical EXOC2 transcript levels. **(a)** Quantitative PCR analysis of EXOC2 transcripts in HEK293T cells stably transduced with the WT (C), variant (G) and the Null (Δ) constructs, respectively. The expression level of EXOC2 was calculated as the fold change of transcript levels in the HEK293T cells transduced with the commercial 3'-UTR reporter (pCS-EGFP-3'). **(b)** Quantitative PCR analysis of EXOC2 transcripts in HEK293T cells stably transduced with tandem repeats of the rs116446171 variant allele. The expression level of EXOC2 was calculated as the fold change of transcript levels in the HEK293T cells transduced with the EGFP vector (pCS-EGFP). In both experiments, the error bars represent standard error of the mean (± s.e.m.) based on 9 replicates. Differences were not significant by the unpaired t-test.

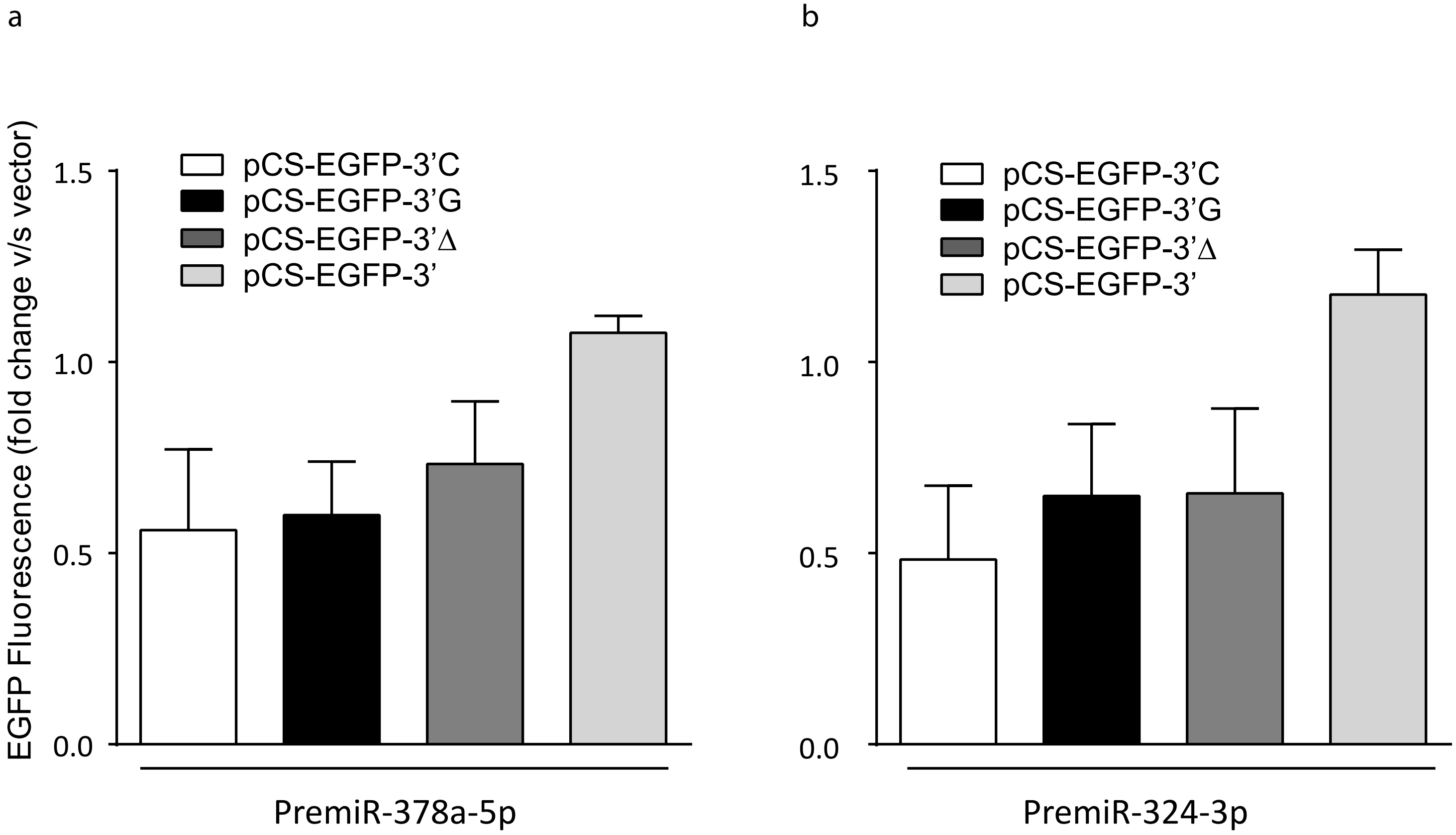

Supplementary Figure 7 | PremiR-378a-5p and PremiR-324-3p effects on EGFP reporter activity. EGFP reporter activity in HEK293T stably transduced cell lines transfected with PremiR-378a-5p **(a)** or PremiR-324-3p **(b)**. Both the PremiR-378a-5p and PremiR-324-3p expression plasmids reduced the EGFP protein expression to a similar extent in cells harboring the wild type (C), variant (G) or null (Δ) construct. There is no decrease of EGFP protein expression in cells harboring the EGFP vector plasmid. The mean ± s.e.m. fold change of EGFP fluorescence for 9 experimental replicates was normalized against the stable cell lines transfected with each PremiR vector respectively.

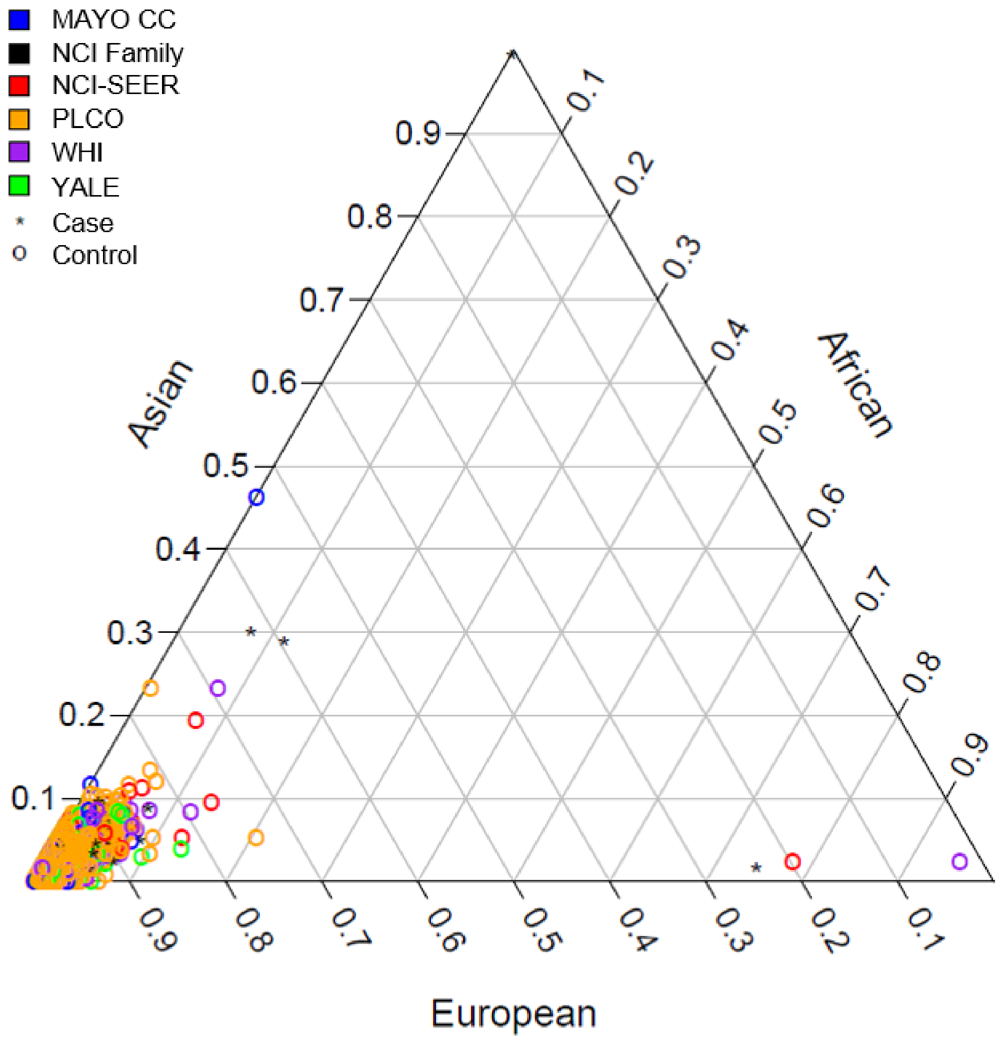

Supplementary Figure 8 | Plot of estimated admixture for individuals in WM/LPL GWAS (Stage 1). For details, see the Methods. Individuals with <80% European ancestry were excluded.

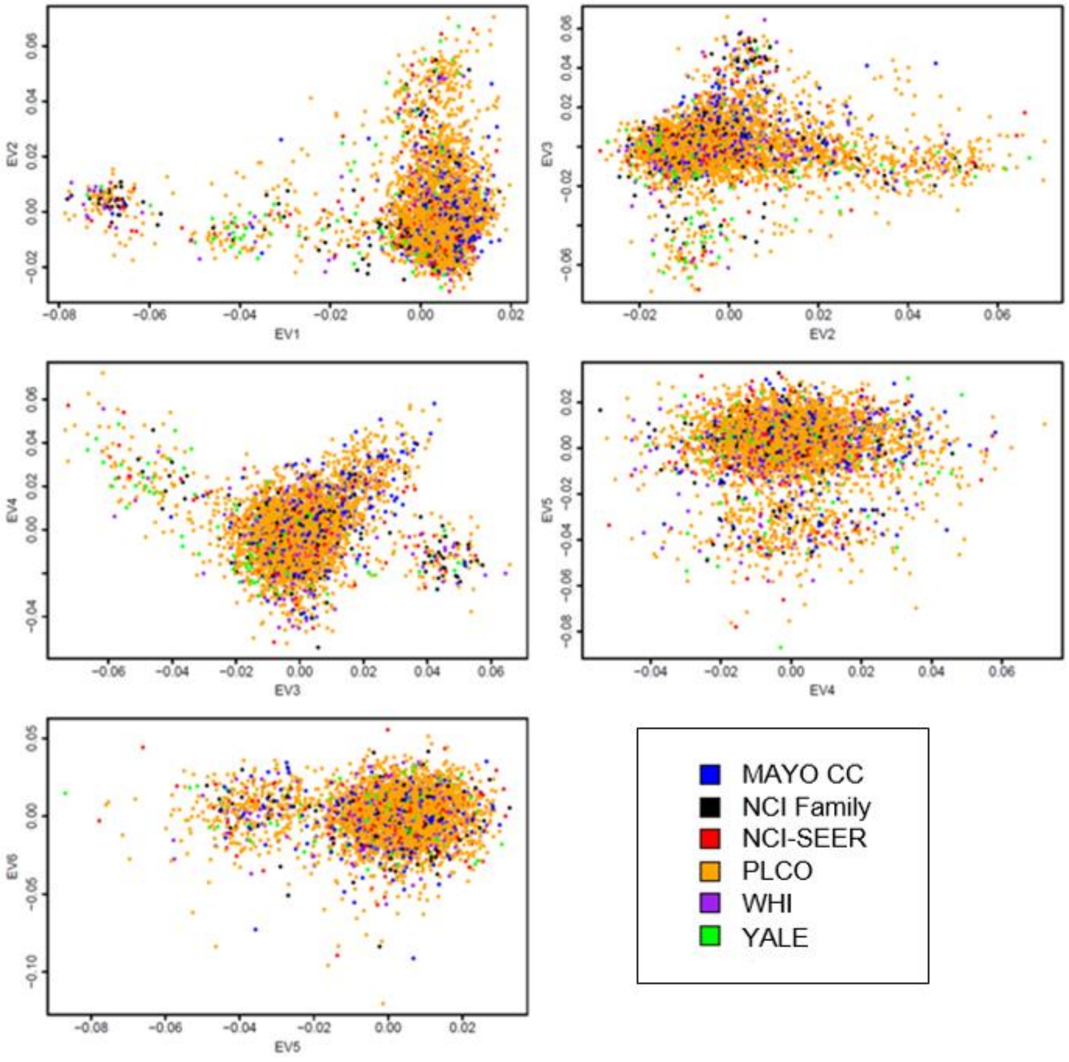

Supplementary Figure 9 | Plot of top five eigenvectors from WM/LPL GWAS (Stage 1) data based on principal components analysis. For details, see Methods.

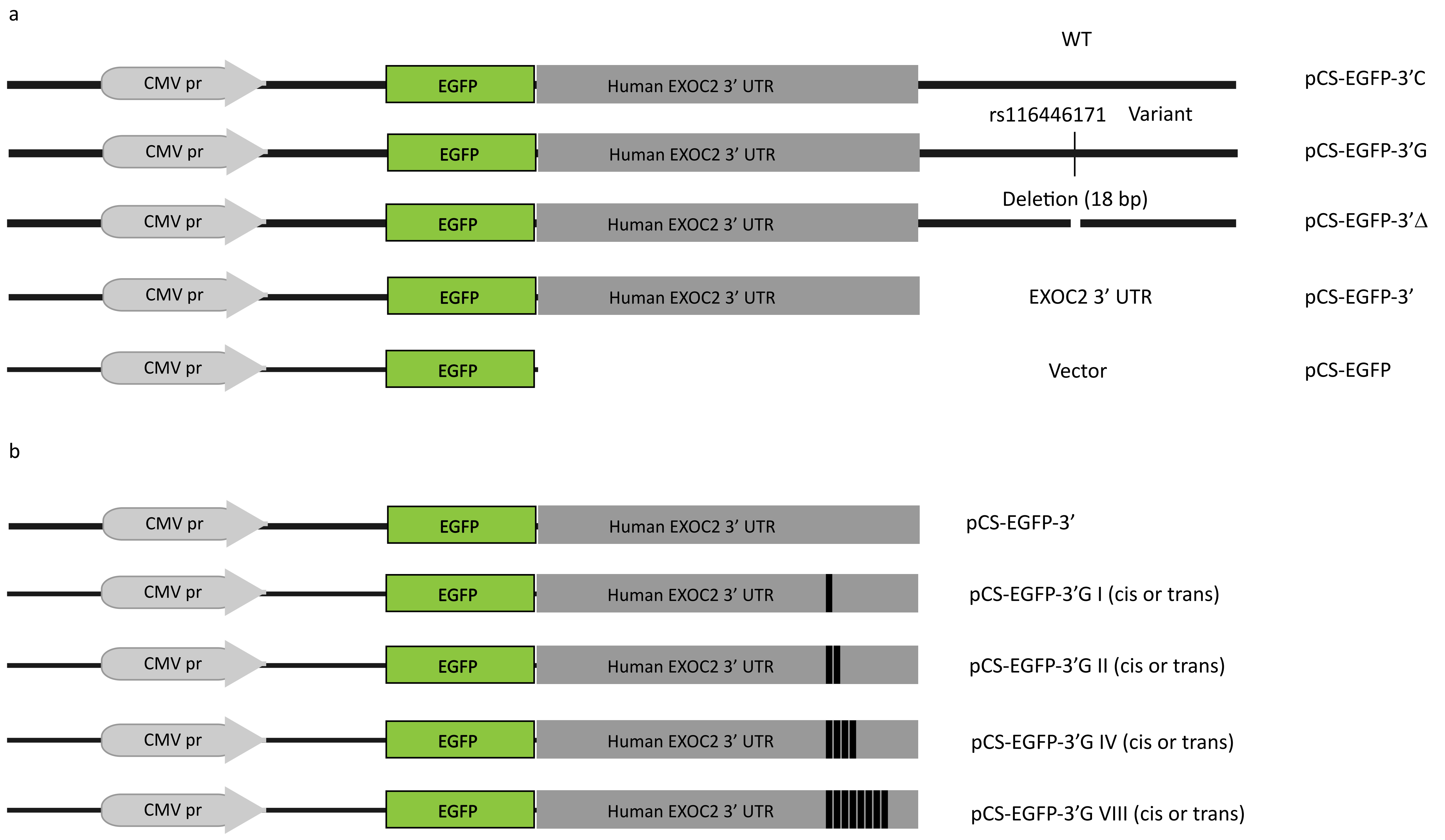

Supplementary Figure 10 | Schematic representations of EGFP reporters constructed for functional expression arrays. Shown are **(a)** the EGFP reporters carrying the extended 3’-UTR of *EXOC2* incorporating rs116446171 and/or deletion of an 18-bp segment centered on rs116446171, the commercial 3’-UTR reporter and the empty vector and **(b)** the EGFP reporters carrying tandem repeats of a 25-bp sequence centered around the rs116446171 variant allele inserted in the 3’-UTR of the *EXOC2* gene.

Supplementary Table 1 | Description and design of studies included in the discovery and replication

Study Name	Study Abbreviation	No. WM/LPL Cases	No. Controls	Design, location	Source of cases	Source of controls	Study Reference
**DISCOVERY**							
**Cases**							
Mayo Clinic Case-Control Study of NHL and CLL	MAYO CC	37	0	Clinic-based case-control study, USA	Consecutive patients with newly diagnosed, histologically-confirmed non-Hodgkin lymphoma (excluding HIV-infected cases) who were residents of Minnesota, Iowa or Wisconsin	N.A.	**[PMID: 21686124]** Cerhan JR, et al. Design and validity of a clinic-based case-control study on the molecular epidemiology of lymphoma. Int J Mol Epidemiol Genet 2011;2(2):95-113.
NCI Study of Individuals and Families at High Risk for Lymphoid and Hematologic Cancers	NCI Family	207	0	Referral-based family cohort study, USA	Cases identified through self-report or physician referral. Verified by medical records and pathology report	N.A.	**[PMID: 20308603]** Royer RH, et al. Differential characteristics of Waldenström macroglobulinemia according to patterns of familial aggregation. Blood 2010;115(22): 4464-4471.
**Previously scanned controls**							
Mayo Clinic Case-Control Study of NHL and CLL	MAYO CC	0	343[a]	Clinic-based case-control study, USA	N.A.	Controls were selected from patients seen in the general medicine clinics at Mayo with a pre-scheduled general medical examination, frequency-matched on age, sex, and geographic region.	**[PMID: 21686124]** Cerhan JR, et al. Design and validity of a clinic-based case-control study on the molecular epidemiology of lymphoma. Int J Mol Epidemiol Genet 2011;2(2):95-113.
National Cancer Institute-Surveillance, Epidemiology, and End Results Interdisciplinary Case-Control Study of Non-Hodgkin's Lymphoma	NCI-SEER	0	270[a]	Population-based case-control study, USA	N.A.	NHL controls ascertained through random digit dialing (<64 years of age) and CMMS files (≥65 years of age)	**[PMID: 15342441]** Chatterjee N, et al. Risk of non-Hodgkin's lymphoma and family history of lymphatic, hematology, and other cancers. CEBP 2004;13:1415-21. **[PMID: 17018637]** Wang SS, et al. Common genetic variants in proinflammatory and other immunoregulatory genes and risk for non-Hodgkin lymphoma. Cancer Res 2006;66(19):9771-80.
Prostate, Lung, Colorectal, and Ovarian Cancer Screening Trial	PLCO	0	2825[b]	Nested case-control, USA	N.A.	Cohort participants without a diagnosis of cancer	**[PMID: 25939597]** Berndt SI, et al. Two susceptibility loci identified for prostate cancer aggressiveness. Nat Commun 2015;6:6889. **[PMID: 16054167]** Hayes RB, et al. Methods for etiologic and early marker investigations in the PLCO trial. Mutat Res 2005;592:147-54.
Women's Health Initiative	WHI	0	228[a]	Nested case-control, USA	N.A.	Cohort participants without a diagnosis of cancer	**[PMID: 14575938]** Anderson GL, et al. Implementation of the Women's Health Initiative study design. Ann Epidemiol 2003 Oct;13(9Suppl):S5-17.
Population-based case-control study in Connecticut women	Yale	0	146[a]	Population-based case-control study, USA	N.A.	Population-based NHL controls ascertained through random digit dialing (<64 years of age) and CMMS files (≥65 years of age)	**[PMID: 19822571]** Zhang Y, et al. Genetic variations in xenobiotic metabolid pathway genes, personal hair dye use and risk of non-Hodgkin lymphoma. Am J Epidemiol 2009;170(10):1222-30.

REPLICATION							
American Cancer Society Cancer Prevention Study-II Nutrition Cohort	CPS-II	39	51	Nested case-control, USA	Self-report through biannual questionnaires (starting in 1997). Verified by medical records or linkage to state cancer registry.	Cohort participants alive at time of case diagnosis without cancer	**[PMID: 11900235]** Calle EE, et al. The American Cancer Society Cancer Prevention Study II Nutrition Cohort: rationale, study design, and baseline characteristics. Cancer 2002;94:2490-2501.
Environmental and genetic risk factors study in adult lymphoma	ENGELA	6	6	Hospital-based case-control study, France	Recent diagnosis of a NHL as per the WHO classification (ICD-O-3) / Cases with AIDS or on immunosuppressant drugs were not eligible. Path reports for 100%, slide review for selected NHL.	Hospitalized in the same hospitals as the cases, for any reason except cancer, an accident or a disease directly related to the subject's occupation, smoking, or alcohol consumption. HIV negative.	**[PMID: 18781390]** Monnereau A, et al. Cigarette smoking, alcohol drinking, and risk of lymphoid neoplasms: results of a French case-control study. Cancer Causes Control. 2008;19(10):1147-1160.
European Prospective Investigation into Cancer, Chronic Diseases, Nutrition and Lifestyles	EPIC	1	1	Nested case-control, multiple European countries	Cases identified through population cancer registries in seven of the participating countries (Denmark, Italy, TheNetherlands, Norway, Spain, Sweden and the UK) and through a combination of methods including health insurance records, cancer and pathology registries, and by active follow-up through study subjects and their next-of-kin in three countries (France, Germany and Greece)	Cohort participants matched by age, sex and study center who were alive and cancer-free at the time of diagnosis of the corresponding case	**[PMID: 9126529]** Riboli E, et al. The EPIC Project: rationale and study design. European Prospective Investigation into Cancer and Nutrition. Int J Epidemiol 1997;26(1):S6-14. **[PMID: 12639222]** Riboli E, et al. European Prospective Investigation into Cancer and Nutrition (EPIC): study populations and data collection. Public Health Nutr 2002;5(68):1113-1124.
Epilymph case-control study in six European countries	EpiLymph	8	8	Multicenter case-control study, hospital-based and population-based, Europe	First primary lymphoma diagnosis according to the 2001 WHO classification of lymphoma	Controls from Germany and Italy were randomly selected by sampling from the general population, matched to cases on gender, 5-year age-group, and residence area. The rest of the centers used matched hospital controls, with eligibility criteria limited to diagnoses other than cancer, infectious diseases and immunodeficient diseases.	**[PMID: 16557575]** Besson H, et al. Tobacco smoking, alcohol drinking and non-Hodgkin's lymphoma: a European multicenter case-control study (Epilymph). Int J Cancer 2006;119:901-8
Health Professionals Follow-up Study	HPFS	5	1	Nested case-control, USA	Self-report through be-annual questionnaires. Verified by medical records and pathology report	Cohort participants alive at time of case diagnosis without cancer, matched on date of birth, ethnicity, date and time of day of blood collection, and fasting status	**[PMID: 1678444]** Rimm E, et al. Prospective study of alcohol consumption and risk of coronary disease in men. Lancet 1991;338:464-468.
Iowa-Mayo SPORE Molecular Epidemiology Resource	Iowa-Mayo SPORE	6	0	Clinic-based case registry, USA	Consecutive patients with newly diagnosed, histologically-confirmed non-Hodgkin lymphoma (excluding HIV-infected cases) who were residents of USA	N.A.	**[PMID: 20713849]** Drake MT, et al. Vitamin D insufficiency and prognosis in non-Hodgkin's lymphoma. J Clin Oncol 2010;28:419104198.

Mayo Clinic Case-Control Study of NHL and CLL	MAYO CC	99	167	Clinic-based case-control study, USA	Consecutive patients with newly diagnosed, histologically-confirmed non-Hodgkin lymphoma (excluding HIV-infected cases) who were residents of Minnesota, Iowa or Wisconsin	Controls were selected from patients seen in the general medicine clinics at Mayo with a pre-scheduled general medical examination, frequency-matched on age, sex, and geographic region.	**[PMID: 21686124]** Cerhan JR, et al. Design and validity of a clinic-based case-control study on the molecular epidemiology of lymphoma. Int J Mol Epidemiol Genet 2011;2(2):95-113.
Memorial-Sloan Kettering Lymphoproliferative disorders study	MSKCC	64	302	Hosptial-based case-study, USA	Hospital clinic based ascertainment in a teritary referral center	NYCP controls from same geographic area	**[PMID: 23349640]** Vijai J, et al. Susceptibility loci associated with specific and shared subtypes of lymphoid malignancies. PLoS Genet 2013;9(1): e1003220.
NCI Study of Individuals and Families at High Risk for Lymphoid and Hematologic Cancers	NCI Family	29	0	Referral-based family cohort study, USA	Cases identified through self-report or physician referral. Verified by medical records and pathology report	N.A.	**[PMID: 20308603]** Royer RH, et al. Differential characteristics of Waldenström macroglobulinemia according to patterns of familial aggregation. Blood 2010;115(22): 4464-4471.
Nurses' Health Study	NHS	4	2	Nested case-control, USA	Self-report through be-annual questionnaires. Verified by medical records and pathology report	Cohort participants alive at time of case diagnosis without cancer, matched on date of birth, ethnicity, date and time of day of blood collection, and fasting status	**[PMID: 15864280]** Colditz GA, et al. The Nurses' Health Study: lifestyle and health among women. Nat Rev Cancer 2005;5:388-396. **[PMID: 7658481]** Hankinson SE, et al. Alcohol, height and adiposity in relation to estrogen and prolactin levels in postmenopausal women. J Natl Cancer Inst 1995;87:1297-1302.
New South Wales non-Hodgkin lymphoma study	NSW	20	20	Population-based case-control study, Australia	Incident NHL diagnosis identified through New South Wales (NSW) or Australian Capital Territory (ACT) cancer registry (excluding HIV-infected cases and transplant recipients)	Controls randomly selected from electoral rolls, matched on age, sex and State of residence at diagnosis.	**[PMID: 15095310]** Hughes AM, et al. Pigmentary characteristics, sun sensitivity and non-Hodgkin lymphoma. Int J Cancer 2004;110:429-434.
Scandinavian Lymphoma Etiology Study	SCALE	26	0	Population-based case-control study, Scandinavia	Patients with incident primary NHL diagnosed through rapid case-ascertainment network in Sweden and Denmark	Frequency matched (age in 10 year intervals, sex and country) population controls prospectively identified every 6 months in nationwide population.	**[PMID: 15687363]** Smedby KE, et al. Ultraviolet radiation exposure and risk of malignant lymphomas. J Natl Cancer Inst 2005;97(3):199-209.
Molecular Epidemiology of non-Hodgkin lymphoma	UCSF2	12	0	Population-based case-control study, USA	RCA/SEER Incident NHL diagnosis for patients diagnosed in hospitals in 6 San Francisco Bay Area Counties and who were residents of the Bay Area at the time of diagnosis	Controls ascertained through RDD were frequency matched to cases on age in 5-year groups, sex and county of residence; Random sampling of CMS lists for person residing in the same 6 Bay Area counties were used to supplement recruitment of controls aged 65+.	[**PMID: 19620980**] Skibola CF, et al. Genetic variants at 6p21.33 are associated with susceptibility to follicular lymphoma. Nat Genet 2009;41(8):873-5. [**PMID: 22697504**] Mikhak B, et al. Intake of vitamins d and a and calcium and risk of non-Hodgkin lymphoma: San Francisco Bay Area population-based case-control study. Nutr Cancer 2012;64(5):674-84.
Population-based case-control study in Connecticut women	Yale	8	8	Population-based case-control study, USA	N.A.	Population-based NHL controls ascertained through random digit dialing (<64 years of age) and CMMS files (≥65 years of age).	**[PMID: 19822571]** Zhang Y, et al. Genetic variations in xenobiotic metabolid pathway genes, personal hair dye use and risk of non-Hodgkin lymphoma. Am J Epidemiol 2009;170(10):1222-30.

[a] *Controls scanned previously on the Illumina OmniExpress for the National Cancer Institute (NCI)/InterLymph Non-Hodgkin lymphoma (NHL) GWAS* [8]

[b] *Controls scanned previously on the Illumina Omni2.5 for the Prostate, Lung, Colorectal, Ovarian (PLCO) cohort* [9]

Supplementary Table 2 | Characteristics of the cases and controls included in the analysis for the discovery and replication

	No. of Subjects		% Male		Mean (SD) Age[a]	
**Study**	**Case**	**Control**	**Case**	**Control**	**Case**	**Control**
**Stage 1 - Discovery**						
NCI Family	182 WM	-	54.4%	-	59.0 (9.9)	-
Mayo Clinic	35 WM	-	74.3%	-	61.1 (13.5)	-
Mayo CC	-	340	-	61.8%	-	60.9 (13.4)
NCI-SEER	-	270	-	54.1%	-	56.6 (12.0)
PLCO	-	2,818	-	100.0%	-	69.6 (6.2)
WHI	-	228	-	0.0%	-	77.7 (6.6)
YALE	-	142	-	0.0%	-	61.7 (13.5)
**Total**	**217 WM**	**3,798**	**57.6%**	**83.6%**	**59.3 (10.5)**	**68.1 (9.3)**
**Stage 2 - Replication**						
NCI Family/InterLymph[b]	66 WM, 104 LPL	117	57.6%	56.4%	65.4 (10.4)	66.7 (10.4)
Mayo CC/SPORE[c]	88 WM	145	72.7%	71.7%	64.5 (10.3)	63.6 (10.9)
MSKCC	45 WM, 10 LPL	302	61.8%	59.6%	61.9 (12.2)	50.6 (10.0)
**Total**	**199 WM, 114 LPL**	**564**	**62.6%**	**62.1%**	**64.5 (10.7)**	**57.3 (12.6)**

[a] *Mean Age is defined as age at diagnosis for cases and age at blood draw or age at selection for controls.*

[b] *Eleven studies in the InterLymph Consortium contributed cases and/or controls (See Methods and Supplementary Table 1).*

[c] *For the replication, Mayo CC/SPORE contributed a total of 99 WM cases and 167 controls (88 cases/145 controls newly ascertained and genotyped at Mayo and 11 cases/22 controls previously contributed to InterLymph and genotyped at NCI. See Methods).*

No., number; SD, standard deviation; WM, Waldenström macroglobulinemia; LPL, lymphoplasmacytic lymphoma; for study abbreviations, see Supplementary Table 1

Supplementary Table 3 | Sequential association analysis of WM-associated SNPs in the 6p25.3 region conditioned on the most significant SNP, rs116446171

SNP_ID	position	info_score	Minor Allele Frequency Cases	Minor Allele Frequency Controls	Unconditional p-value*	P-value after conditioning on rs116446171
rs2671429	394266	0.9916	0.0792	0.0330	2.28E-08	0.8780
rs2797307	394293	0.9964	0.0792	0.0326	1.58E-08	0.8555
rs2671428	394643	0.9950	0.0792	0.0325	1.62E-08	0.8552
rs2797308	394711	0.9944	0.0815	0.0325	3.45E-09	0.6905
rs78518495	426758	0.9934	0.1872	0.1016	2.22E-09	0.1765
rs563341023	427717	0.9752	0.0724	0.0117	1.51E-23	0.7519
rs950286	429457	0.9978	0.1878	0.1015	1.73E-09	0.1656
rs138522854	440106	0.9702	0.0725	0.0117	1.21E-23	0.7216
rs6914942	441630	0.9993	0.1923	0.1017	1.15E-09	0.2293
rs10900949	445278	0.9959	0.1922	0.1021	1.02E-09	0.2207
rs1533117	445706	0.7326	0.0301	0.0429	0.0449	0.2765
rs1533117	445706	0.9867	0.1995	0.1090	2.74E-09	0.1926
rs1533121	446400	0.9952	0.1924	0.1022	9.72E-10	0.2206
rs57706053	451909	0.9515	0.0748	0.0137	9.12E-20	0.8353
rs112137351	455779	0.9539	0.0743	0.0144	9.88E-19	0.6330
rs73374968	456173	0.9538	0.0742	0.0143	7.56E-19	0.6324
rs72833984	466544	0.9399	0.0683	0.0106	8.40E-21	0.9275
rs13205238	468597	0.9703	0.2121	0.0930	2.78E-15	0.8390
rs9405705	470384	0.9323	0.2188	0.1202	1.88E-09	0.1625
rs9503944	471435	0.9949	0.1842	0.0681	8.30E-13	0.1493
rs13219910	471619	0.9546	0.2504	0.1309	1.39E-08	0.8574
rs9378846	471640	0.9763	0.2373	0.1115	6.17E-10	0.9756
rs73376906	471669	0.9750	0.2346	0.1079	4.87E-10	0.9800
rs73376966	471719	0.9764	0.2347	0.1077	3.65E-10	0.9680
rs4959926	472484	0.9868	0.2398	0.1141	1.84E-09	0.9993
rs4959927	472723	0.9894	0.2399	0.1149	2.83E-09	0.9666
rs4959928	472807	0.9912	0.2400	0.1146	2.72E-09	0.9770
rs6597036	473148	0.9947	0.2432	0.1185	3.46E-09	0.9971
rs6597037	473248	0.9982	0.2399	0.1143	2.36E-09	0.9762
rs9392029	474252	0.9996	0.2489	0.1201	2.32E-09	0.8143
rs9503977	475154	0.9989	0.2628	0.1350	1.20E-08	0.7845
rs9503978	475246	0.9827	0.2566	0.1277	2.86E-08	0.6914
rs34926067	475249	0.9825	0.2566	0.1277	2.87E-08	0.6917
rs73376981	475649	0.9926	0.2497	0.1204	1.39E-09	0.7783
rs4959273	475810	0.9892	0.2473	0.1176	3.50E-10	0.7991
rs143730329	477568	0.9853	0.0783	0.0105	7.22E-28	0.4255
rs12333255	479361	0.9869	0.0808	0.0105	2.58E-28	0.5562
rs76106586	483593	0.9945	0.1605	0.0193	6.43E-52	0.9999
rs116446171*	484453	0.9981	0.1606	0.0191	4.88E-52	0.9881
rs9392616	512071	0.9942	0.0815	0.0152	2.48E-19	0.7893
rs7761186	512951	0.9961	0.0815	0.0154	3.50E-19	0.7794
rs7760561	513175	0.9992	0.0815	0.0153	2.81E-19	0.7889
rs7776401	513279	0.9958	0.0816	0.0155	3.45E-19	0.7758
rs541373128	521135	0.9894	0.0781	0.0125	4.89E-26	0.6784
rs2064302	556106	0.9670	0.0909	0.0305	7.31E-10	0.1209
rs3765438	563210	0.9791	0.0907	0.0306	8.39E-10	0.1226
rs75402334	575613	0.9043	0.0661	0.0106	2.12E-17	0.2316
rs180743203	664246	0.9006	0.0569	0.0128	8.89E-15	0.2729
rs141155157	748474	0.8590	0.0444	0.0118	1.38E-09	0.6227

**Association analysis results before conditioning on the most significant WM/LPL-associated SNP, rs116446171*

P-values generated using a log-additive genetic model, adjusting for age, sex and significant principal components.

Supplementary Table 4 | Linkage disequilibrium (r^2) among potential WM/LPL-associated SNPs within the 14q32.13-2 region

SNP_ID	rs179159	rs117089797	rs117484046	rs113907803	rs117410836	rs142581138	rs117593121	rs59462958	rs10145930	rs112494872	rs55762860
rs179159	1	0.132	0.132	0.132	0.002	0.004	0.002	0	0.006	0.001	0.03
rs117089797	0.132	1	1	1	0.006	0.001	0.001	0.001	0	0	0.002
rs117484046	0.132	1	1	1	0.006	0.001	0.001	0.001	0	0	0.002
rs113907803	0.132	1	1	1	0.006	0.001	0.001	0.001	0	0	0.002
rs117410836	0.002	0.006	0.006	0.006	1	0.504	0.385	0.38	0.016	0.008	0
rs142581138	0.004	0.001	0.001	0.001	0.504	1	0.838	0.774	0.027	0.003	0
rs117593121	0.002	0.001	0.001	0.001	0.385	0.838	1	0.631	0.03	0.005	0
rs59462958	0	0.001	0.001	0.001	0.38	0.774	0.631	1	0.031	0.003	0
rs10145930	0.006	0	0	0	0.016	0.027	0.03	0.031	1	0	0.003
rs112494872	0.001	0	0	0	0.008	0.003	0.005	0.003	0	1	0.219
rs55762860	0.03	0.002	0.002	0.002	0	0	0	0	0.003	0.219	1

Increasing intensity of shading corresponds to increasing degree of linkage disequilibrium (r^2).

Supplementary Table 5 | Sequential conditional analysis of 14q32.13-2 SNPs conditioned on the most significant SNPs in apparently independent linkage disequilibrium blocks

SNP_ID	Position	info_score	Minor Allele Frequency (MAF)		Unconditional p-value	P-value after conditioning on rs117410836*	P-value after conditioning on rs117410836 and rs179159†
			Cases	Controls			
rs179159†	95972448		0.3620	0.2471	4.50E-07	3.59E-07	0.9435
rs117089797	95995282	0.9413	0.1525	0.0739	2.92E-09	2.69E-05	0.0431
rs117484046	95995283	0.9416	0.1525	0.0739	2.93E-09	2.70E-05	0.0431
rs113907803	95995666	0.9431	0.1525	0.0740	3.15E-09	2.88E-05	0.0440
rs117410836*	96051974	0.9459	0.1146	0.0262	3.12E-19	0.4109	0.4422
rs142581138	96072463	0.9527	0.1287	0.0488	1.91E-10	0.8777	0.9465
rs117593121	96076744	0.9456	0.1232	0.0452	2.19E-10	0.4171	0.4577
rs59462958	96098192	0.9584	0.1242	0.0479	4.06E-10	0.4824	0.5639
rs10145930	96130955		0.1945	0.1149	3.01E-05	2.12E-04	5.28E-03
rs112494872	96154477	0.9583	0.0704	0.0292	4.61E-07	9.54E-04	9.49E-04
rs55762860	96171808	0.9828	0.0965	0.0458	4.80E-07	2.14E-04	2.50E-03

** Association model results after conditioning on the most significant WM/LPL-associated SNP, rs117410836, in the LD block containing rs117410836, rs142581138, rs117593121, and rs59462958 (See Supplementary Table 4).*

† Association model results after additive conditioning on the two most significant WM/LPL-associated SNPs, rs117410836 and rs179159. SNPs rs117089797, rs117484046 and rs113907803 constitute a third significant LD block while rs10145930, rs1124982 and rs55762860 are each independent from each other and all other nearby LD blocks (See Supplementary Table 4).

P-values were generated using a log-additive genetic model, adjusting for age, sex and two principal components.

Supplementary Table 6 | Association with WM or WM/LPL for all SNPs taken forward for replication

SNP	Chr	Position[a]	Effect allele[b]	Other allele	Stage	Genotyped /Imputed[c]	EAF[d]	Controls (n)	WM only Cases (n)	WM only OR	WM only (95% CI)	WM only *P*	WM/LPL Cases (n)	WM/LPL OR	WM/LPL (95% CI)	WM/LPL *P*
rs116446171	6	484453	G	C	Discovery	I (0.9985)	0.019	3,798	217	56.44	(32.89-96.85)	1.59E-48	217	56.44	(32.89-96.85)	1.59E-48
					Replication	G	0.020	564	198	9.38	(5.27-16.71)	2.86E-14	312	7.71	(4.46-13.33)	2.54E-13
					**Combined**			**4,362**	**415**	**24.41**	**(16.46-36.23)**	**7.43E-57**	**529**	**21.14**	**(14.40-31.03)**	**1.36E-54**
rs117410836	14	96051974	C	T	Discovery	I (0.9611)	0.027	3,798	217	10.62	(6.17-18.29)	1.63E-17	217	10.62	(6.17-18.29)	1.63E-17
					Replication	G	0.036	563	197	2.78	(1.69-4.59)	0.0001	306	2.81	(1.77-4.45)	1.16E-05
					**Combined**			**4,361**	**414**	**5.14**	**(3.56-7.43)**	**2.78E-18**	**523**	**4.90**	**(3.45-6.96)**	**8.75E-19**
rs7760561	6	513175	G	A	Discovery	G	0.015	3,798	217	24.27	(12.26-48.06)	5.64E-20	217	24.27	(12.26-48.06)	5.64E-20
					Replication	G	0.021	564	199	2.93	(1.61-5.32)	0.0004	312	2.66	(1.50-4.69)	0.0008
					**Combined**			**4,362**	**416**	**7.31**	**(4.66-11.45)**	**3.96E-18**	**529**	**6.57**	**(4.24-10.17)**	**3.17E-17**
rs179159_60[e]	14	95972448	A	G	Discovery	G	0.247	3,798	217	1.79	(1.42-2.26)	6.88E-07	217	1.79	(1.42-2.26)	6.88E-07
					Replication	G	0.250	564	199	1.39	(1.06-1.81)	0.0166	312	1.29	(1.02-1.65)	0.0369
					**Combined**			**4,362**	**416**	**1.61**	**(1.35-1.91)**	**1.04E-07**	**529**	**1.54**	**(1.30-1.81)**	**4.66E-07**
rs1600403	3	140128682	G	A	Discovery	I (0.9914)	0.038	3,798	217	5.62	(3.21-9.84)	1.5E-09	217	5.62	(3.21-9.84)	1.50E-09
					Replication	G	0.033	563	198	0.86	(0.44-1.68)	0.6514	311	0.97	(0.54-1.76)	0.9324
					**Combined**			**4,361**	**415**	**2.61**	**(1.70-4.01)**	**1.30E-05**	**528**	**2.45**	**(1.64-3.69)**	**1.49E-05**
rs2826333	21	21867435	G	A	Discovery	G	0.197	3,798	217	1.87	(1.46-2.40)	7.67E-07	217	1.87	(1.46-2.40)	7.67E-07
					Replication	G	0.194	563	199	1.10	(0.82-1.48)	0.5068	311	1.15	(0.89-1.49)	0.2890
					**Combined**			**4,361**	**416**	**1.50**	**(1.24-1.82)**	**2.67E-05**	**528**	**1.48**	**(1.24-1.78)**	**1.63E-05**
rs4748248	10	16135267	T	A	Discovery	I (0.9989)	0.664	3,798	217	1.73	(1.39-2.15)	7.41E-07	217	1.73	(1.39-2.15)	7.41E-07
					Replication	G	0.681	445	133	0.92	(0.68-1.26)	0.6211	143	0.97	(0.72-1.32)	0.8523
					**Combined**			**4,243**	**350**	**1.41**	**(1.18-1.69)**	**0.0002**	**360**	**1.42**	**(1.19-1.70)**	**8.49E-05**
rs79354307	16	6735531	T	C	Discovery	I (0.9944)	0.028	3,798	217	4.86	(2.70-8.75)	1.34E-07	217	4.86	(2.70-8.75)	1.34E-07
					Replication	G	0.028	563	199	0.93	(0.44-1.94)	0.8371	312	0.97	(0.51-1.82)	0.9135
					**Combined**			**4,361**	**416**	**2.57**	**(1.62-4.07)**	**6.07E-05**	**529**	**2.30**	**(1.49-3.53)**	**0.0002**
rs13297645	9	34422185	A	G	Discovery	G	0.405	3,798	217	1.79	(1.46-2.21)	4.09E-08	217	1.79	(1.46-2.21)	4.09E-08
					Replication	G	0.401	562	199	0.93	(0.73-1.19)	0.5771	312	0.95	(0.77-1.18)	0.6594
					**Combined**			**4,360**	**416**	**1.36**	**(1.16-1.59)**	**0.0001**	**529**	**1.32**	**(1.14-1.54)**	**0.0003**
rs2249099	6	30079307	A	C	Discovery	G	0.143	3,798	217	2.10	(1.56-2.83)	9.66E-07	217	2.10	(1.56-2.83)	9.66E-07
					Replication	G	0.155	561	199	0.84	(0.59-1.18)	0.3026	311	0.97	(0.72-1.29)	0.8161
					**Combined**			**4,359**	**416**	**1.41**	**(1.13-1.77)**	**0.0025**	**528**	**1.41**	**(1.15-1.74)**	**0.0011**
rs77128578	9	34545655	C	A	Discovery	I (0.7735)	0.019	3,798	217	13.37	(4.61-38.75)	1.78E-06	217	13.37	(4.61-38.75)	1.78E-06
					Replication	G	0.021	563	199	0.35	(0.10-1.22)	0.0986	311	0.73	(0.32-1.66)	0.4580
					**Combined**			**4,361**	**416**	**2.90**	**(1.29-6.53)**	**0.0102**	**528**	**2.16**	**(1.13-4.14)**	**0.0198**

[a] *Position according to human reference NCBI37/hg19*

[b] *Allele associated with an effect on risk for WM/LPL*

[c] *G=genotyped; I=imputed (info score)*

[d] *Effect allele frequency in controls*

[e] *Results shown are combined for rs179159 and rs179160, which are in high LD (r^2= 0.96). In the Stage 2 replication rs179159 was genotyped by one center using Taqman and rs179160 was genotyped by two centers using Sequenom (see Methods).*

P-values and odds ratios were generated using a log-additive genetic model, adjusting for age, sex and two principal components. The stage 1 discovery analysis was adjusted for age, sex and significant principal components. The stage 2 replication analysis was adjusted for age, sex, Ashkenazi Jewish status and genotyping center. The combined meta-analysis used a fixed-effects model.

WM, Waldenström macroglobulinemia; LPL, lymphoplasmacytic lymphoma; Chr, chromosome; EAF, effect allele frequency; OR, odds ratio; CI, confidence interval; info, information; LD, linkage disequilibrium

Supplementary Table 7 | Technical validation concordance rates for eleven SNPs genotyped in WM/LPL GWAS replication

SNP	Chr	Concordant Genotype Calls (n)	Total Genotype Calls (n)	Concordance Rate
rs116446171	6p25.3	679	680	0.999
rs117410836	14q32.13	665	670	0.993
rs1600403	3q23	682	685	0.996
rs2249099	6p21.3	680	680	1.000
rs7760561	6p25.3	686	687	0.999
rs13297645	9p13	686	686	1.000
rs77128578	9p13	670	688	0.974
rs10904678	10p13	684	686	0.997
rs179159	14q32.13	681	682	0.999
rs79354307	16p13.3	684	686	0.997
rs2826333	21q21	689	689	1.000
Overall		7486	7519	0.996

SNP, single nucleotide polymorphism; WM, Waldenström macroglobulinemia; LPL, lymphoplasmacytic lymphoma; Chr, chromosome

Supplementary Table 8 | Frequency of the corresponding variant allele in affected relatives of index WM/LPL cases having either the rs116446171 or rs117410836 variant allele

	Affected 1st-degree relatives				Affected >1st-degree relatives				Total affected relatives			
**Condition**	**Mean age at diagnosis (yrs)**	**No. with effect allele (n)**	**No. cases related to index cases (n)**	**%**	**Mean age at diagnosis (yrs)**	**No. with effect allele (n)**	**No. cases related to index cases (n)**	**%**	**Mean age at diagnosis (yrs)**	**No. with effect allele (n)**	**No. cases related to index cases (n)**	**%**
**Affected relatives of 17 index cases having the rs116446171 effect allele (G)**												
WM	59.6	7	10	70	68.1	3	4	75	62.0	10	14	71
IgM MGUS	65.3	6	7	86	61.0	3	3	100	64.0	9	10	90
Other LPD	61.7	2	3	67	61.0	1	3	33	61.3	3	6	50
Total	61.9	15	20	75	63.9	7	10	70	62.6	22	30	73
**Affected relatives of 13 index cases having the rs117410836 effect allele (C)**												
WM	55.9	7	9	78	70.0	3	3	100	59.0	10	12	83
IgM MGUS	55.8	4	4	100	61.0	1	1	100	56.8	5	5	100
Other LPD	71.0	1	3	33	30.0	1	1	100	60.8	2	4	33
Total	58.7	12	16	75	60.2	5	5	100	59.0	17	21	81

Among WM/LPL cases ("index cases") in the discovery stage, 17 cases with the rs116446171 (G) variant allele and 13 cases with the rs117410836 (C) variant allele also had relatives who were diagnosed ("affected") with either WM, IgM MGUS, or a related LPD and who had genotyping data available for analysis (See Methods). Note that 5 index cases had the effect allele at both SNPs and are included in both groups.

No., number; WM, Waldenström macroglobulinemia; MGUS, monoclonal gammopathy of undetermined significance; LPD, lymphoproliferative disorder

Supplementary Table 9 | Adjusted Genotype-Tissue Expression Project (GTEx) data to assess presence of rs116446171 *cis-* eQTL in cells derived from EBV-transformed lymphocytes and whole blood*

Chr	Gene_Ensembl_ID	Gene_name	Gene_start	Gene_end	Gene_type	Distance to rs116446171 (bp)	N	*P* -value	Beta	SE
**EBV-transformed lymphocytes**										
6	ENSG00000112685.9	EXOC2	485133	693117	protein_coding	680	337	0.8534	-0.0498	0.2690
6	ENSG00000230433.1	RP1-20B11.2	524171	525581	antisense	39718	337	0.0633	-0.5054	0.2711
6	ENSG00000137265.10	IRF4	391739	411447	protein_coding	73006	337	0.7899	0.0711	0.2665
6	ENSG00000112679.10	DUSP22	291630	351355	protein_coding	133098	337	0.4162	0.2174	0.2671
6	ENSG00000188996.4	HUS1B	655939	656963	protein_coding	171486	337	0.3297	-0.2538	0.2599
6	ENSG00000272463.1	RP11-532F6.3	708592	711405	lincRNA	224139	337	0.9632	0.0125	0.2697
6	ENSG00000164379.4	FOXQ1	1312675	1314992	protein_coding	828222	337	0.4573	-0.1974	0.2652
6	ENSG00000272279.1	RP11-157J24.2	1528599	1529146	lincRNA	1044146	337	0.3954	0.2275	0.2672
6	ENSG00000054598.5	FOXC1	1610681	1614127	protein_coding	1126228	337	0.1375	-0.4087	0.2745
6	ENSG00000112699.6	GMDS	1624041	2245926	protein_coding	1139588	337	0.1454	-0.3881	0.2658
6	ENSG00000250903.4	GMDS-AS1	2245982	2482256	lincRNA	1761529	337	0.9187	0.0277	0.2709
6	ENSG00000272465.1	RP1-136B1.1	2437783	2438483	lincRNA	1953330	337	0.4631	0.1987	0.2705
**Whole blood**										
6	ENSG00000112685.9	EXOC2	485133	693117	protein_coding	680	113	0.1553	-1.0502	0.7329
6	ENSG00000230433.1	RP1-20B11.2	524171	525581	antisense	39718	113	0.3207	-0.7370	0.7380
6	ENSG00000137265.10	IRF4	391739	411447	protein_coding	73006	113	0.4503	0.5651	0.7454
6	ENSG00000112679.10	DUSP22	291630	351355	protein_coding	133098	113	0.0063	-2.0130	0.7202
6	ENSG00000188996.4	HUS1B	655939	656963	protein_coding	171486	113	0.6335	0.3753	0.7844
6	ENSG00000272463.1	RP11-532F6.3	708592	711405	lincRNA	224139	113	0.5514	-0.4466	0.7470
6	ENSG00000263667.1	RP3-416J7.4	181466	205484	lincRNA	278969	113	0.4156	-0.5954	0.7280
6	ENSG00000054598.5	FOXC1	1610681	1614127	protein_coding	1126228	113	0.1449	1.0894	0.7407
6	ENSG00000112699.6	GMDS	1624041	2245926	protein_coding	1139588	113	0.0094	1.8579	0.7003
6	ENSG00000250903.4	GMDS-AS1	2245982	2482256	lincRNA	1761529	113	0.8324	0.1574	0.7418
6	ENSG00000236086.3	HMGN2P28	2341684	2341950	pseudogene	1857231	113	0.1732	1.0522	0.7664
6	ENSG00000272465.1	RP1-136B1.1	2437783	2438483	lincRNA	1953330	113	0.1235	-1.0954	0.7045

** Data are shown for genes within 2 megabases (Mb) of rs116446171*

P-values generated using the Wald test

eQTL, expression quantitative trait loci; EBV, Epstein-Barr virus; bp, base pairs; SE, standard error

Supplementary Table 10 | Stage 1 and Stage 2 subjects genotyped, quality control exclusions, and subjects included in the analysis

	Genotyped Subjects		Exclusions						Final subjects included in analysis	
			Diagnostic	High	Gender					
Study	Cases	Controls	misclassification	missing rate	discordance	Duplicates	Relatedness	Non CEU	Cases	Controls
**Stage 1 Discovery**										
**Cases**										
NCI Family	207	-	5	7	0	2	9	2	182	-
Mayo Clinic	37	-	0	0	0	0	0	2	35	-
**Total**	**244**	**-**	**5**	**7**	**0**	**2**	**9**	**4**	**217**	
**Previously scanned controls**[a]										
Mayo CC	-	343	-	0	0	0	0	3	-	340
NCI-SEER	-	270	-	0	0	0	0	0	-	270
PLCO	-	2825	-	0	0	1	4	2	-	2818
WHI	-	228	-	0	0	0	0	0	-	228
YALE	-	146	-	0	0	0	0	4	-	142
Total	-	3812	-	0	0	1	4	9	-	3798
**Grand total**			**5**	**7**	**0**	**3**	**13**	**13**	**217**	**3798**
**Stage 2 Replication**										
**Cases**										
NCI Family/InterLymph	170	-	0	0	0	0	0	0	170	-
Mayo CC/SPORE	96	-	0	0	0	0	0	8[b]	88	-
MKSCC	58	-	3	0	0	0	0	0	55	-
**Total**	324	-	3	0	0	0	0	0	313	-
**Controls**										
NCI Family/InterLymph	-	117	0	0	0	0	0	0	-	117
Mayo CC/SPORE	-	146	0	0	0	0	0	1[b]	-	145
MSKCC	-	302	0	0	0	0	0	0	-	302
**Total**	-	565	0	0	0	0	0	0	-	564
**Grand total**			**3**	**0**	**0**	**0**	**0**	**9**	**313**	**564**

[a] *Controls scanned previously as part of the NCI/InterLymph NHL GWAS* [8] *and PLCO* [9] *.*

[b] *In the replication, 8 cases and 1 control were excluded because race was unknown.*

CEU, Utah residents with Northern and Western European ancestry from the CEPH collection; for study abbreviations, see Supplementary Table 1

Supplementary Table 11 | Distribution of genotypes for top WM/LPL SNPs across cases and controls

SNP	Effect allele/ Other allele	Genotype	Discovery				Replication			
			No. of cases	No. of controls	OR	(95% CI)	No. of cases	No. of controls	OR	(95% CI)
**rs116446171**	G/C	CC	152	3,654	1.00	—	241	542	1.00	—
		CG	61	143	10.24	(7.29-14.41)	68	22	7.58	(4.36-13.19)
		GG	4	1	95.98	(10.66-864.26)	3	0	NA	NA
**rs117410836**	C/T	TT	171	3,597	1.00	—	258	525	1.00	—
		CT	44	200	4.68	(3.26-6.70)	43	36	3.01	(1.79-5.04)
		CC	2	1	35.91	(3.65-353.14)	5	2	5.00	(0.85-29.42)

WM, Waldenström macroglobulinemia; LPL, lymphoplasmacytic lymphoma; SNP, single nucleotide polymorphism; No., number; OR, odds ratio; CI, confidence interval

Supplementary Note 1

ACKNOWLEDGEMENTS

Support for individual studies: CPS-II (L.R.T.) —The American Cancer Society funds the creation, maintenance and updating of the CPS-II cohort. We thank the CPS-II participants and the Study Management Group for their invaluable contributions to this research. We would also like to acknowledge the contribution to this study from the central cancer registries supported through the Centers for Disease Control and Prevention National Program of Cancer Registries and cancer registries supported by the National Cancer Institute Surveillance Epidemiology and End Results program; ELCCS (A.S.) – Bloodwise; ENGELA (A.M.) – Fondation ARC pour la Recherche sur le Cancer, Fondation de France, French Agency for Food, Environmental and Occupational Health & Safety (ANSES), the French National Cancer Institute (INCa); EPIC – Coordinated Action (Contract #006438, SP23-CT-2005-06438), HuGeF (Human Genetics Foundation), Torino, Italy; EpiLymph – European Commission (grant references QLK4-CT-2000-00422 and FOOD-CT-2006-023103), the Spanish Ministry of Health (grant references CIBERESP, PI14/01219, RCESP C03/09, RTICESP C03/10 and RTIC RD06/0020/0095), the Marató de TV3 Foundation (grant reference 051210), the Agència de Gestió d' Ajuts Universitaris de Recerca—Generalitat de Catalunya (grant reference 2014SGR756) who had no role in the data collection, analysis or interpretation of the results; the NIH (contract NO1-CO-12400); the Compagnia di San Paolo—Programma Oncologia; the Federal Office for Radiation Protection grants StSch4261 and StSch4420, the José Carreras Leukemia Foundation grant DJCLS-R12/23, the German Federal Ministry for Education and Research (BMBF-01-EO-1303); the Health Research Board, Ireland and Cancer Research Ireland; Czech Republic supported by MH CZ—DRO (MMCI, 00209805) and by the project MEYS – NPS I – LO1413; Fondation de France and Association de Recherche Contre le Cancer; HPFS (Walter C. Willet) – The HPFS was supported in part by National Institutes of Health grants CA167552, CA149445 and CA098122. We would like to thank the participants and staff of the Health Professionals Follow-up Study for their valuable contributions as well as the following state cancer registries for their help: AL, AZ, AR, CA, CO, CT, DE, FL, GA, ID, IL, IN, IA, KY, LA, ME, MD, MA, MI, NE, NH, NJ, NY, NC, ND, OH, OK, OR, PA, RI, SC, TN, TX, VA, WA, WY. The authors assume full responsibility for analyses and interpretation of these data; Iowa-Mayo SPORE (J.R.C.) – National Institutes of Health (CA97274), Specialized Programs of Research Excellence (SPORE) in Human Cancer (P50 CA97274), Molecular Epidemiology of Non-Hodgkin

Lymphoma Survival (R01 CA129539), Henry J. Predolin Foundation; Mayo Clinic Case-Control (J.R.C.) – National Institutes of Health (R01 CA92153), National Center for Advancing Translational Science (UL1 TR000135), Mayo Clinic Cancer Center Support grant P30 CA15083; MSKCC (K.O.) – Geoffrey Beene Cancer Research Grant, Lymphoma Foundation (LF5541), Barbara K. Lipman Lymphoma Research Fund (74419), Robert and Kate Niehaus Clinical Cancer Genetics Research Initiative (57470), U01 HG007033; NCI Family (M.L.M., L.R.G., N.E.C.) – Intramural Research Program of the National Cancer Institute, National Institutes of Health, DHHS (02-C-0210). We would like to thank our participants for their invaluable contributions; NCI-SEER – Intramural Research Program of the National Cancer Institute, National Institutes of Health, and Public Health Service (N01-PC-65064, N01-PC-67008, N01-PC-67009, N01-PC-67010, N02-PC-71105); NHS (Meir J. Stampfer) – The NHS was supported in part by National Institutes of Health grants CA186107, CA87969, CA49449, CA149445 and CA098122. We would like to thank the participants and staff of the Nurses' Health Study for their valuable contributions as well as the following state cancer registries for their help: AL, AZ, AR, CA, CO, CT, DE, FL, GA, ID, IL, IN, IA, KY, LA, ME, MD, MA, MI, NE, NH, NJ, NY, NC, ND, OH, OK, OR, PA, RI, SC, TN, TX, VA, WA, WY. The authors assume full responsibility for analyses and interpretation of these data; NSW (C.M.V.) – was supported by grants from the Australian National Health and Medical Research Council (ID990920), the Cancer Council NSW, and the University of Sydney Faculty of Medicine; PLCO – This research was supported by the Intramural Research Program of the National Cancer Institute and by contracts from the Division of Cancer Prevention, National Cancer Institute, NIH, DHHS; SCALE (K.E.S., H.H.) – Swedish Cancer Society (2009/659), Stockholm County Council (20110209) and the Strategic Research Program in Epidemiology at Karolinska Institute, Swedish Cancer Society grant (02 6661), Danish Cancer Research Foundation Grant, Lundbeck Foundation Grant (R19-A2364), Danish Cancer Society Grant (DP 08-155), National Institutes of Health (5R01 CA69669-02), Plan Denmark; UAB (C.F.S., A.I.O.) – NIH R01 (CA154643 and CA104682; C.F.S.), V Foundation for Cancer Research (A.I.O.); UCSF2 (C.F.S., P.M.B.) – This study was supported by the NCI, National Institutes of Health, grants CA1046282 and CA154643 (C.F.S.), and R01CA87014, R01CA45614, R03CA14397, and R03CA150037 (P.M.B). The collection of cancer incidence data used in this study was supported by the California Department of Health Services as part of the statewide cancer reporting program mandated by California Health and Safety Code Section 103885; the National Cancer Institute's Surveillance, Epidemiology, and End Results Program under contract HHSN261201000140C awarded to the Cancer Prevention Institute of California, contract HHSN261201000035C awarded to the University of Southern California, and contract HHSN261201000034C awarded to the Public Health Institute; and the Centers for Disease Control and

Prevention's National Program of Cancer Registries, under agreement #1U58 DP000807-01 awarded to the Public Health Institute. The ideas and opinions expressed herein are those of the authors, and endorsement by the State of California, the California Department of Health Services, the National Cancer Institute, or the Centers for Disease Control and Prevention or their contractors and subcontractors is not intended nor should be inferred; WHI — The WHI program is funded by the National Heart, Lung, and Blood Institute, National Institutes of Health, U.S. Department of Health and Human Services through contracts HHSN268201600018C, HHSN268201600001C, HHSN268201600002C, HHSN268201600003C, and HHSN268201600004C." The authors thank the WHI investigators and staff for their dedication, and the study participants for making the program possible. A full listing of WHI investigators can be found at: http://www.whi.org/researchers/Documents%20%20Write%20a%20Paper/WHI%20Investigator%20Long%20List.pdf; YALE (Y.Z.) – National Cancer Institute (CA62006).; YALE (Y.Z.) – National Cancer Institute (CA62006).

SUPPLEMENTARY REFERENCES

1. Javierre, B.M. *et al.* Lineage-specific genome architecture links enhancers and non-coding disease variants to target gene promoters. *Cell* **167**, 1369-84 (2016).
2. Jin, F. *et al.* A high-resolution map of the three-dimensional chromatin interactome in human cells. *Nature* **503**, 290-4 (2013).
3. Cairns, J. *et al.* CHiCAGO: robust detection of DNA looping interactions in Capture Hi-C data. *Genome Biol.* **17**, 127 (2016).
4. Zhang, Y. *et al.* Model-based analysis of ChIP-Seq (MACS). *Genome Biol.* **9**, R137 (2008).
5. Kasowski, M. *et al.* Extensive variation in chromatin states across humans. *Science* **342**, 750-2 (2013).
6. Roadmap Epigenomics Consortium *et al.* Integrative analysis of 111 reference human epigenomes. *Nature* **518**, 317-30 (2015).
7. ENCODE Project Consortium. An integrated encyclopedia of DNA elements in the human genome. *Nature* **489**, 57-74 (2012).
8. Berndt, S.I. *et al.* Genome-wide association study identifies multiple risk loci for chronic lymphocytic leukemia. *Nat. Genet*. **45**, 868-876 (2013).
9. Berndt, S.I. *et al.* Two susceptibility loci identified for prostate cancer aggressiveness. *Nat. Commun*. **6**, 6889 (2015).
